# Supplementary material for: Evidence that the TRPV1 S1-S4 membrane domain contributes to thermosensing
Source: Nat Commun. 2020 Aug 20;11:4169. doi: 10.1038/s41467-020-18026-2 (PMC7441067; doi:10.1038/s41467-020-18026-2)
Supplement: Supplementary file 1 — Supplementary Information [file 41467_2020_18026_MOESM1_ESM.pdf]

Supplementary Information

Evidence that the TRPV1 S1-S4 Membrane Domain Contributes to Thermosensing

Minjoo Kim[1,2,†], Nicholas J. Sisco[1,2,†], Jacob K. Hilton[1,2], Camila M. Montano[2], Manuel A. Castro[1], Brian R. Cherry[3], Marcia Levitus[1,4], Wade D. Van Horn[1,2*]

Affiliations:

[1]School of Molecular Sciences, Arizona State University, 551 E. University Drive, Tempe, AZ. 85287.

[2]The Biodesign Institute Virginia G. Piper Center for Personalized Diagnostics, Arizona State University, Tempe, AZ. 85287.

[3]The Magnetic Resonance Research Center, Arizona State University, Tempe, AZ. 85287.

[4]The Biodesign Institute Center for Single Molecule Biophysics, Arizona State University, Tempe, AZ. 85287.

[†]These Authors contributed equally to this work.

[*]Correspondence to: wade.van.horn@asu.edu

Contents

Supplementary Methods

Details of the thermodynamic framework for interpreting temperature-dependent studies

Simulation of the temperature dependence of NMR peak height

Supplementary Figures

Supplementary Figure 1: The expression, purification, and NMR detergent screening of the hV1-S1S4

Supplementary Figure 2: NMR amide backbone experiment strips of the hV1-S1S4 S2-S3 loop and the S4 helix

Supplementary Figure 3: The amide backbone NMR assignments of the hV1-S1S4

Supplementary Figure 4: Capsaicin binding induces widespread hV1-S1S4 chemical shift perturbation

Supplementary Figure 5: Temperature-dependent TRPV1 and hV1-S1S4 data

Supplementary Figure 6: Additional data supporting the thermodynamic analyses of the hV1-S1S4 using various biophysical methods

Supplementary Figure 7: Simulation of the temperature dependence of NMR peak height parameters

Supplementary Figure 8: Supporting data for RDC and PRE NMR measurements

Supplementary Figure 9: Comparison of hV1-S1S4 ^{1}H-^{15}N RDCs with values predicted from the rTRPV1 cryo-EM structure

Supplementary Figure 10: Primary amide proton temperature coefficient data and residual chemical shift analysis

Supplementary Figure 11: Mapping the TRPV1 S1-S4 residues that show temperature-dependent movement

Supplementary Figure 12. Further characterization of R557A hTRPV1 and the R557A hV1-S1S4 domain in temperature and capsaicin activation

Supplementary Figure 13: hTRPV1 R557A is a functional proton activated channel

Supplementary Figure 14: Structural examples of S1-S4 motions in TRP channel activation

Supplementary Figure 15: Putative temperature-dependent motions in the TRPV3 S1-S4 domain

Supplementary Tables

Supplementary Table 1: Measured thermosensitivity values (ΔH) from TRPV1 temperature studies

Supplementary Table 2: Buffer pH stability as a function of temperature

Supplementary Table 3: Comparison between ^{1}H-^{15}N RDCs of hV1-S1S4 and values predicted from cryo-EM structures of TRPV1 and TRPV3.

Supplementary Table 4: TRPV1 DNA primers.

Supplementary Table 5: Parameters used to simulate the temperature dependence of NMR peak height.

Supplementary Methods

Details of the thermodynamic framework for interpreting temperature-dependent studies

NMR resonance intensities are inversely proportional to the resonance linewidth ($\Delta\upsilon_{FWHH}$, full-width at half-height). For the Lorentzian shaped signals observed in NMR, the intensities and linewidths reflect the transverse relaxation rates (R_2) of the molecule, which can be estimated from the linewidth, $\Delta\upsilon_{FWHH} = R_2/\pi$ [1]. R_2 is in turn sensitive to the dynamic properties of the protein in solution, including nanosecond timescale rotational motion, and picosecond to millisecond internal motions, including protein conformational change. Thus, NMR peak intensity should report on the conformational state of a protein. In the hV1-S1S4 temperature-dependent NMR studies, we leverage the common framework employed in CD to assess the thermosensitivity in terms of change in enthalpy (*ΔH*) as described below.

Far-UV CD is commonly used to measure the change in enthalpy (ΔH) between folded or conformational states [2]. This assumes that the change in measured ellipticity as a function of temperature is directly proportional to the change in protein states. Early studies have shown for two-state behavior that there is good correlation between calorimetric and CD measurements of thermodynamic values (c.f.) [3]. To evaluate the ΔH for the transition between protein states, one generally assumes a two-state model. In the

context of TRP channels this is certainly an oversimplification, but adequately captures accurate thermodynamic values, as has been noted in previous studies [4]. Briefly, for temperature-dependent equilibrium, CD data at a given wavelength, usually at 222 nm for helical proteins, exhibit clear changes between two states (i.e. the data are sigmoidal in nature). At a given temperature the relative concentrations of conformational states 1 and 2 will be related to the equilibrium constant K, which can be written as the fraction of the concentration of conformational state 2 (α):

$$K = \frac{[State\ 2]}{[State\ 1]} = \frac{\alpha}{1-\alpha} \qquad [1]$$

K is also related to the change in standard state free energy ($\Delta G°$) between conformational states and noted in the Gibbs-Helmholtz equation:

$$\Delta G° = -RT\ ln\ K \qquad [2]$$

which is, in turn, is related to the changes in enthalpy ($\Delta H°$) and entropy ($\Delta S°$) between states:

$$\Delta G° = \Delta H° - T\Delta S° \qquad [3]$$

At the midpoint of the transition between temperature-dependent conformational states (T_{50}), or the melting temperature (T_m) in folding studies, the concentrations of the two structural states are equal and $K = 1$ and $\Delta G° = 0$, resulting in the following identity:

$$\Delta S° = \frac{\Delta H°}{T_{50}} \qquad [4]$$

Combining equations 2, 3, and 4, K can be written in a modified form of the van't Hoff equation using only terms of temperature (T), midpoint temperature (T_{50}), and the change in enthalpy ($\Delta H°$):

$$K = e^{\left[\left(\frac{\Delta H°}{RT}\right)\left(\left(\frac{T}{T_{50}}\right)-1\right)\right]} \qquad [5]$$

Rearrangement of Equation 1 gives:

$$\alpha = \frac{K}{(1+K)} \qquad [6]$$

A mathematical description of the assumption above, that the observed ellipticity at a given temperature (θ_{obs}) is directly proportional to the ellipticity of conformational states 1 (θ_1) and 2 (θ_2) gives the following relationship:

$$\theta_{obs} = \alpha(\theta_1 - \theta_2) + \theta_1 \qquad [7]$$

To calculate the values of ΔH and T_{50} that best describe the experimental data, initial estimates of ΔH, T_{50}, θ_1, and θ_2 are used as initial parameters to non-linear least-squares fitting of Equation 5, 6, and 7 to the raw data. The authors used SigmaPlot to perform non-linear least squared fitting of the CD data which resulted in a $\Delta H = 19 \pm 1$ kcal/mol and $T_{50} = 37 \pm 1$ °C.

The method described above assumes that change in the heat capacity (ΔC_p) is negligible. In the context of TRP channel thermosensors, it has been suggested that this may not be the case [5]. Indeed, Chanda and

coworkers have used protein design principles that attempt to increase the magnitude of ΔC_p for the Shaker voltage-gated potassium channel, a non-thermosensing ion channel, to successfully convert it to a thermosensing ion channel [6]. Chanda's study clearly indicates that modifying the change in heat capacity can perturb the thermosensitivity of a given protein. However, for wild-type TRP channels at biologically relevant temperatures, it seems that ΔC_p is relatively small in magnitude. When ΔC_p changes as a function of temperature then both ΔH and ΔS will change significantly as a function of temperature [5,7]. However, as reviewed elsewhere [8], existing studies of TRP channel thermosensitivity suggest that ΔH (and thus ΔS) are fairly constant as a function of biologically relevant temperature indicating that ΔC_p is small in magnitude [4,8-12].

The above described method is completely general for extracting ΔH from equilibrium data that can be approximated by a simple two-state model. To this end, these same procedures were used to estimate the ΔH from temperature-dependent whole-cell patch-clamp electrophysiology steady-state currents (Fig. 2A), where θ_1, and θ_2 were modified to maximal and minimal current values. The resulting electrophysiology values are consistent with previously published TRPV1 thermodynamic values (Table S1). Similarly, NMR resonance intensity as a function of temperature exhibits two-state behavior which was also fit to the above equilibrium thermodynamic model, yielding a ΔH value that is similar in magnitude to the CD determined value from the hV1-S1S4 (Fig. 2B and fig. S3E). Lastly, this general two-state equation was also used to interpret the temperature-dependence of the intrinsic tryptophan fluorescence in terms of the average emission wavelength ($\langle\lambda\rangle$), which yielded values of hV1-S1S4 that are consistent with both NMR and CD.

Simulation of the temperature dependence of NMR peak height.

A simplified evaluation of NMR resonance peak height, one that that omits signal loss during data acquisition, data processing and similar, depends on transverse relaxation rate, R_2, which in turn depends on the rotational correlation time, τ_c. The rotational correlation time is also influenced by temperature, in part because of a dependence on the viscosity of water which varies depending on temperature. The following will provide a theoretical framework to estimate NMR peak height as a function of temperature.

The viscosity of water as a function of temperature can be estimated from the following equation:

$$\eta_{H_2O}(T) = A \times 10^{\left(\frac{B}{T-C}\right)} \quad [8]$$

Where the coefficients have the following values: $A \approx 2.414 \times 10^{-5}\ Pa \cdot s$, $B \approx 247.8\ K$, and $C \approx 140\ K$. The resulting water viscosity values agree well with experimental values determined at discreet temperatures[13].

The rotational correlation time (τ_c) impacts NMR resonance lineshape and intensity. It is directly proportional to the viscosity of water and inversely proportional to temperature as noted by the Stokes-Einstein equation. These relationships lead to temperature dependence of the rate constant for tumbling:

$$\tau_c = \frac{4\pi \cdot \eta_{H_2O} \cdot r_H^3}{3k_{\mathrm{B}} \cdot T} \quad [9]$$

The measurable effect that τ_c has on NMR resonance lineshape and intensity can be modeled using the spectral density function. The spectral density function, $J(\omega)$, for a rigid protein, (i.e, the order parameter

$S^2 = 1$) and negligible internal molecular motion ($\tau_e = 0$), gives rise to a simplified version of the Lipari-Szabo Model-Free spectral density function[14,15]:

$$J(\omega) = \frac{\tau_c}{1 + (\omega\tau_c)^2} \quad [10]$$

For solution NMR of biomolecules, spectral sensitivity and resolution are dominated by the transverse relaxation rate. The transverse relaxation rate can be estimated from the spectral density function evaluated at distinct frequencies. In this context, omega (ω) is a frequency of either ^1H or ^{15}N. Evaluation of the spectral density function at several frequencies gives rise to estimates of NMR relaxation rate constants, including the transverse relaxation rate constant, R_2. Parameterizing the frequencies most relevant to this study with values reflected in our experimental studies gives rise to the following:

$$J(0) = \frac{\tau_c}{1 + (0 \cdot \tau_c)^2} = \tau_c \quad [11]$$

$$J(\omega_H) = \frac{\tau_c}{1 + (\omega_H\tau_c)^2} = \frac{\tau_c}{1 + (850.279 \times 10^6\, Hz \cdot \tau_c)^2} \quad [12]$$

$$J(\omega_N) = \frac{\tau_c}{1 + (\omega_N\tau_c)^2} = \frac{\tau_c}{1 + (86.168 \times 10^6\, Hz \cdot \tau_c)^2} \quad [13]$$

The transverse relaxation rate, R_2, can be estimated from the transverse relaxation rate contributions from amide proton ^{15}N dipole-dipole relaxation ($R_{2,DD}$) according to:

$$R_{2,DD} = \frac{a}{8}[4J(0) + 3J(\omega_N) + J(\omega_H - \omega_N) + 6J(\omega_H) + 6J(\omega_H + \omega_N)] \quad [14]$$

And the ^{15}N chemical shift anisotropy ($R_{2,CSA}$):

$$R_{2,CSA} = \frac{b}{6}[4J(0) + 3J(\omega_N)] \quad [15]$$

Such that the sum of these contributions for an ^1H-^{15}N pair in an HSQC-type experiment can be estimated as follows:

$$R_2 = R_{2,DD} + R_{2,CSA} \quad [16]$$

Which when written explicitly results in the following expression:

$$R_2 = \frac{a}{8}[4J(0) + 3J(\omega_N) + J(\omega_H - \omega_N) + 6J(\omega_H) + 6J(\omega_H + \omega_N)] + \frac{b}{6}[4J(0) + 3J(\omega_N)] \quad [17]$$

Where $a = \frac{\mu_0^2\hbar^2\gamma_H^2\gamma_N^2}{16\pi^2 r_{HN}^6}$ and $b = \frac{\Delta\sigma^2\omega_N^2}{3}$, and the meanings and values for the variables used in a and b are listed in Table 4.

The transverse relaxation rate is related to the resonance linewidth, also known as the full-width at half-height, $\Delta\nu_{FWHH}$ as follows:

$$R_2 = \pi\Delta\nu_{FWHH} \quad [18]$$

For a normalized Lorentzian lineshape, the intensity as a function of frequency, $I(\nu)$, can be defined in terms of the center frequency of the Lorentzian, ν_0 the frequency of interest, ν and the linewidth, $\Delta\nu_{FWHH}$ as follows:

$$I(\nu) = \frac{1}{2\pi}\frac{\Delta\nu_{FWHH}}{(\nu-\nu_0)^2+\left(\frac{\Delta\nu_{FWHH}}{2}\right)^2} \quad [19]$$

The Lorentzian maximum, i.e. peak height, occurs where at the center frequency such that:

$$I_{max} = \frac{\Delta\nu_{FWHH}}{2\pi\left(\frac{\Delta\nu_{FWHH}}{2}\right)^2} = \frac{2}{\pi\Delta\nu_{FWHH}} \quad [20]$$

Taken together, the peak height (I_{max}) of a Lorentzian NMR resonance is inversely proportional to the linewidth ($\Delta\nu_{FWHH}$) and directly proportional to R_2, as seen by combining eqns. 18 and 20. This theoretical framework allows for one to estimate the expected relationship between temperature and an amide HSQC resonance peak height (I_{max}) in the limits of a rigid protein and under ideal experimental conditions. Given that we experimentally measured the rotational correlation times for hV1-S1S4 at 20 and 50 °C, to best interpret the PRE distance measurements, we can refine our theoretical intensity model by using the Stokes-Einstein equation (shown above, Eqn. 9) to estimate the Stokes radius of hydration, r_H, for hV1-S1S4 embedded in LPPG micelles assuming a hard sphere. The average r_H, value from these correlation times is 36.5 Å. Similarly, since our measurements were performed on an NMR spectrometer (19.97 T) with a nominal ^{1}H frequency of 850 MHz, we can leverage this information to simulate the idealized peak height dependence on temperature for hV1-S1S4 using values listed in Table 4. Ultimately, the generally sigmoidal nature of the peak height detected from hV1-S1S4 HSQC experiments differ from that of a non-thermosensing and rigid protein as simulated here (Supplementary Fig. 7), which supports the data from orthogonal circular dichroism and fluorescence measurements, and reinforces our conclusion that the hV1-S1S4 participates in TRPV1 thermosensing and temperature-dependent gating.

Supplementary Figures

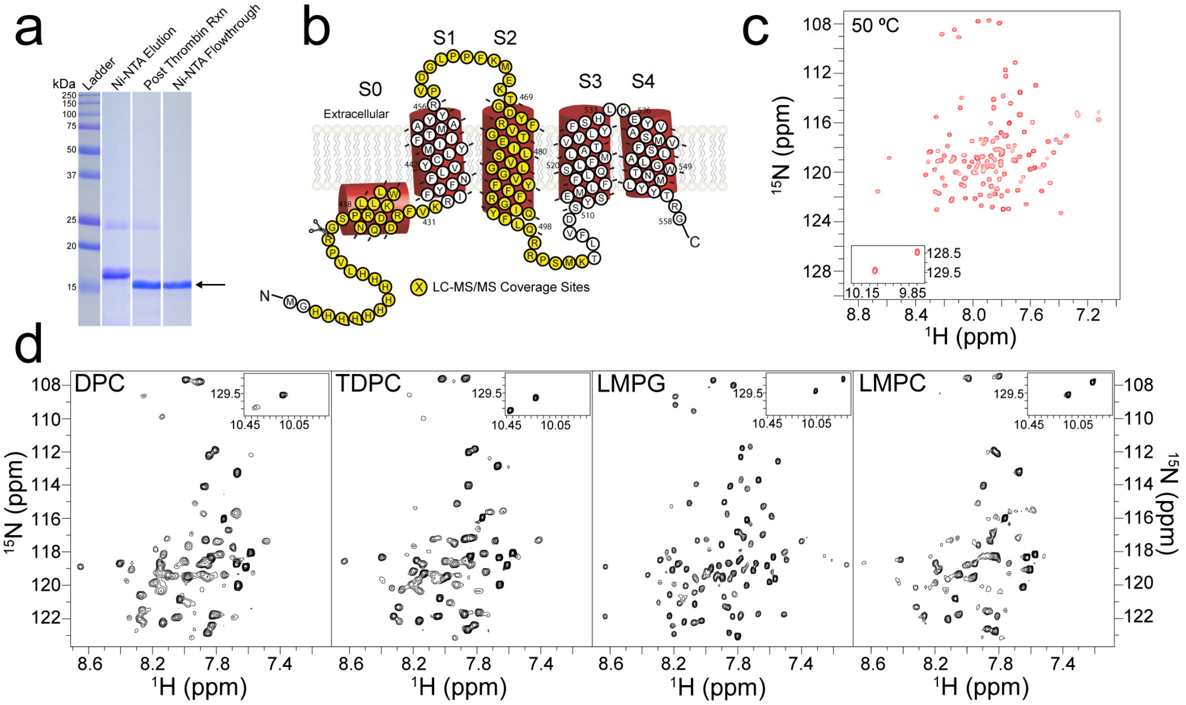

Supplementary Figure 1. The expression, purification, and NMR detergent screening of the hV1-S1S4. (a) SDS-PAGE gel of the hV1-S1S4 samples of Ni^{2+}-NTA affinity chromatography purification, post-thrombin reaction, and 10×His-tag cleaved hV1-S1S4 (lanes 2, 3, and 4 respectively). (**b**) The highlighted residues on the hV1-S1S4 membrane topology represent the coverage for LC-MS/MS as a means to verify the identity of hV1-S1S4 (51% amino acid coverage). (**c**) hV1-S1S4 reconstituted in LPPG micelles results in a high quality ^{1}H-^{15}N TROSY-HSQC NMR spectrum at elevated temperatures (50 °C). (**d**) Optimization of the micellar conditions for the hV1-S1S4 using NMR. To identify a suitable membrane mimic for hV1-S1S4, five membrane mimics were tested. LPPG was chosen to be the most suitable membrane mimic for the hV1-S1S4 as shown in (**c**) after analyzing the number of resonances observed, spectral resolution, and homogeneity of resonance intensities. Source data are provided as a Source Data file.

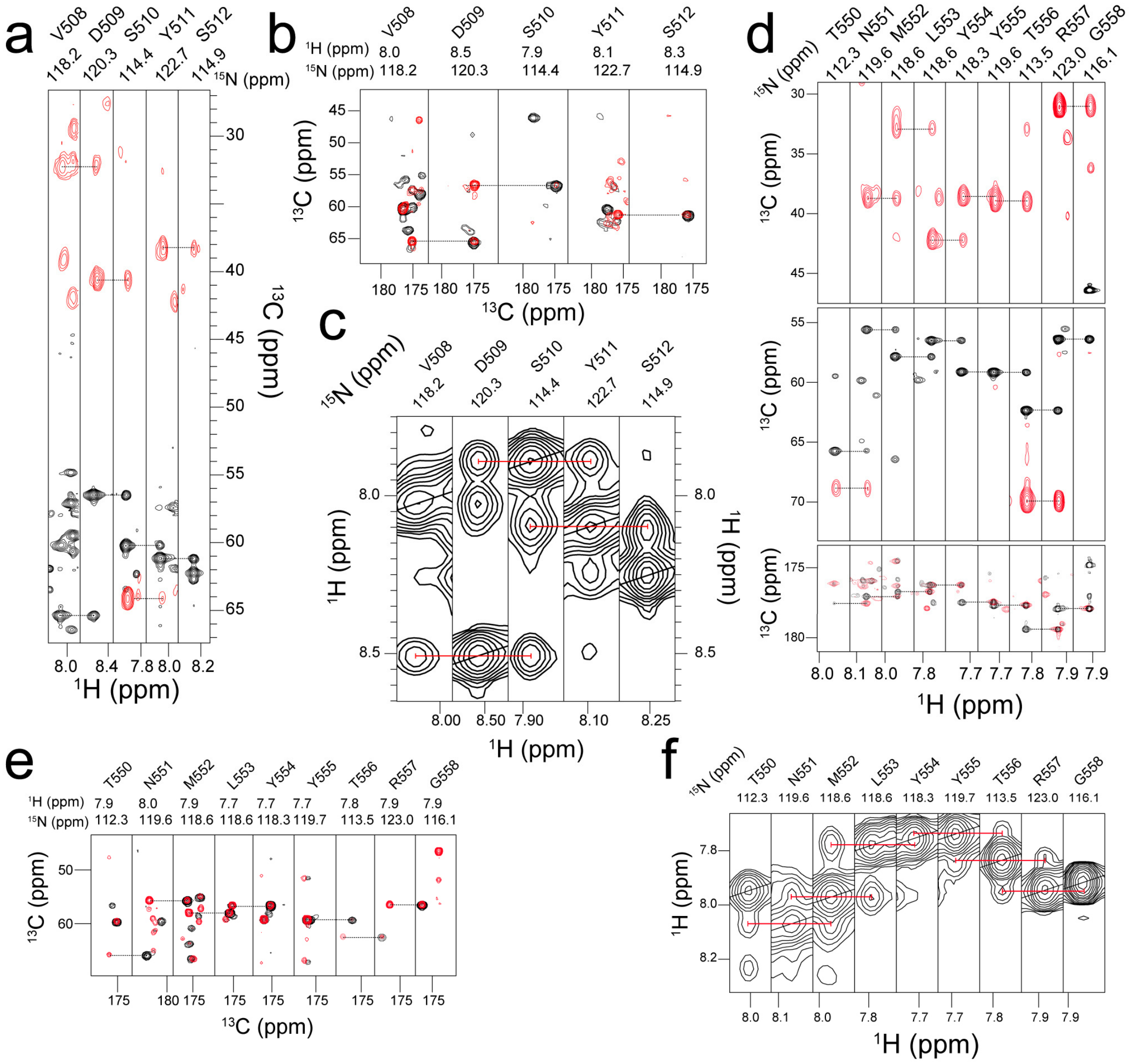

Supplementary Figure 2. NMR amide backbone experiment strips of the hV1-S1S4 S2-S3 loop and the S4 helix. (**a**) Residues in the TRPV1 S2-S3 loop have been implicated in vanilloid ligand binding and activation. A strip plot of HNCA and HNCOCA from V508 to S512 in the S2-S3 loop is shown on the left (**a**), and this assignment is confirmed by the 4D HNCA and HNCOCA (**b**) and ^{15}N-edited NOESY-TROSY (**c**). (**d**) Backbone resonance assignment of T550 to G558 in S4 helix, shown with HNCA (black) and HNCACB (red), 4D HNCACO (**e**, black), and ^{15}N-edited NOESY-TROSY (**f**). Assignments have been deposited in the BMRB database, entry 27029.

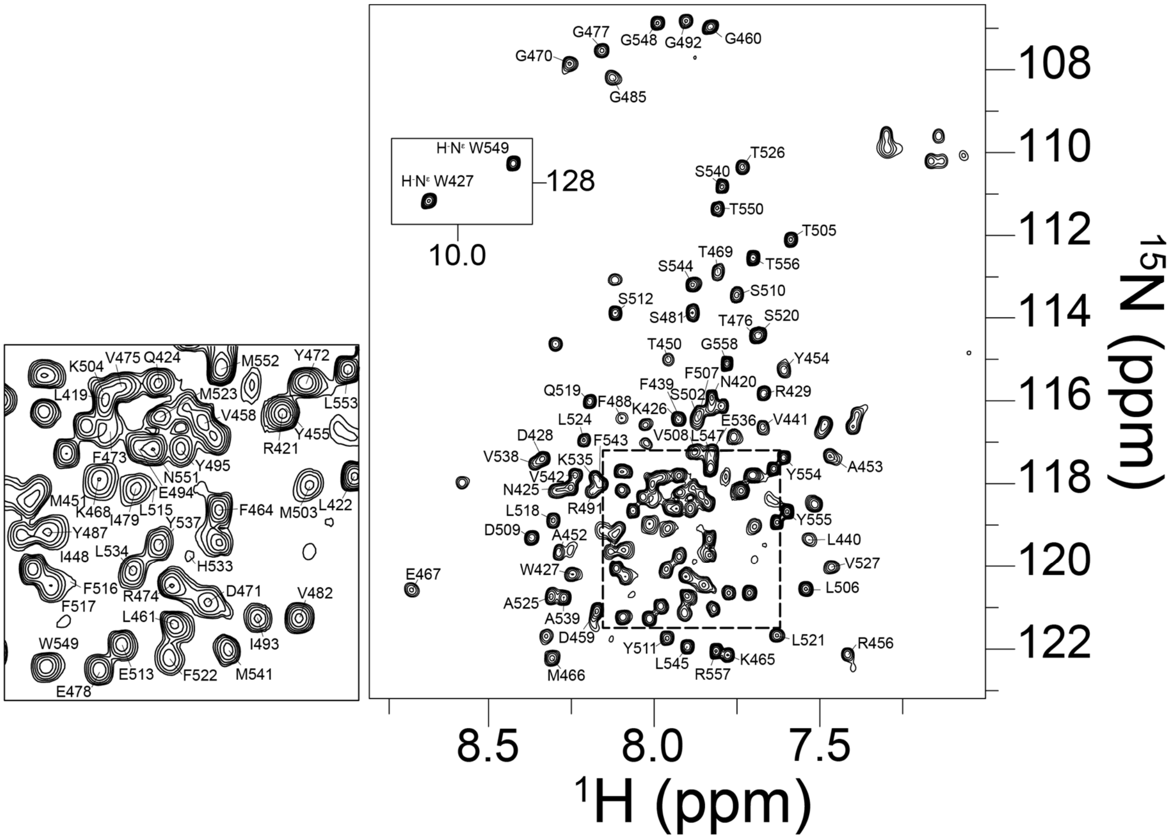

Supplementary Figure 3. The amide backbone NMR resonance assignments of the hV1-S1S4. Approximately 87% of the amide backbone has been confidently assigned. The dashed box in the spectral panel is expanded to the left, highlighting a crowded spectral region in the ^{1}H-^{15}N TROSY HSQC spectrum.

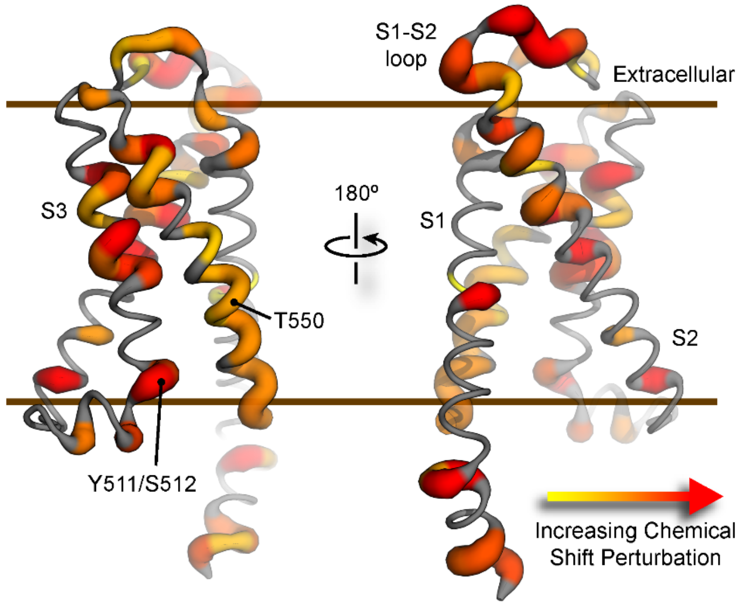

Supplementary Figure 4. Capsaicin binding induces widespread hV1-S1S4 chemical shift perturbation. Capsaicin-induced chemical shift perturbations (CSPs) mapped onto the rat TRPV1 cryo-EM structure (5irz) S1-S4 domain indicate that capsaicin binding impacts regions beyond the canonical binding site. The binding site is highlighted by residues Y511, S512, and T550 in the S3 and S4 helices, respectively (left panel). Of note is that the S1-S2 loop experiences significant CSP which suggests allosteric coupling. Zheng and coworkers have identified that the S1-S2 loop interacts with divalent cations to potentiate TRPV1 for gating [16,17]. Specifically, these interactions with the S1-S2 loop increase both capsaicin and heat activation. This data is from Figure 1d with increasing CSP shown as a gradient from yellow to red. Gray regions are unassigned at 37 °C in the presence of capsaicin. Source data are provided as a Source Data file.

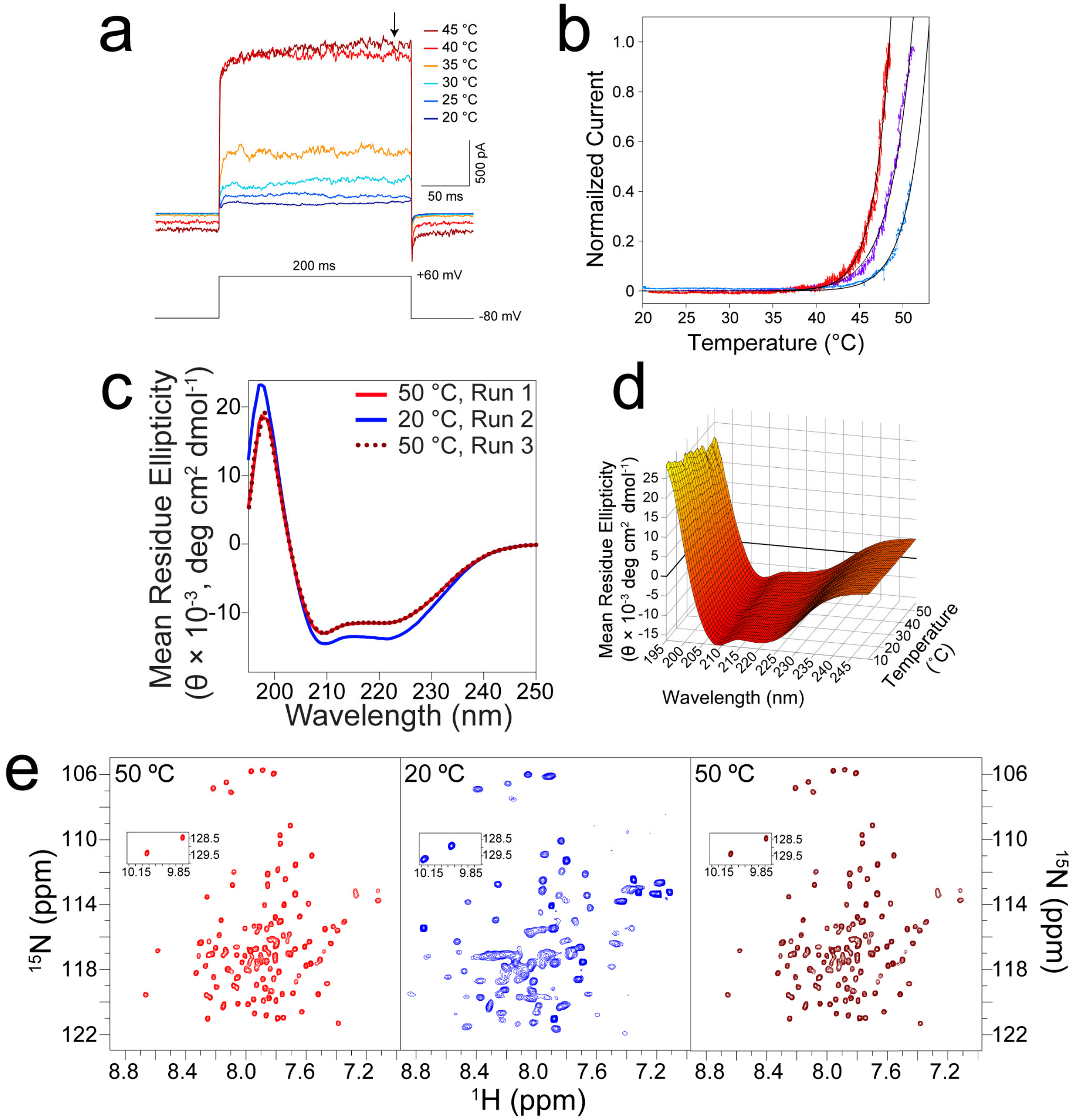

Supplementary Figure 5. Temperature-dependent TRPV1 and hV1-S1S4 data. (**a**) Thermodynamic measurements of human TRPV1 using electrophysiology. Increased temperature evoked increased current magnitudes, and the data points at the black arrow were plotted against the temperature to generate the plot in Fig. 2A. (**b**) Three individual temperature ramp current values obtained from HEK-293 cells transiently transfected with hTRPV1. Cells were subjected to a temperature ramp from 20 to 50 °C over the course of ca. 35 seconds. The ΔH values from each line (red, purple and teal) are obtained from fitting to a pseudo-steady state model (see Methods), resulting in values of 106.8 ± 0.4, 80.1 ± 0.3, and 94.3 ± 0.5 kcal/mol, respectively. (**c**) The reversibility of the hV1-S1S4 was tested with CD between 20 °C and 50 °C. The CD spectra fully recover the initial mean residue ellipticity when re-heated. The data shown are of a temperature cycle of elevated, decreased, and returned elevated temperature spectra. (**d**) Superimposed circular dichroism spectra from 10 °C to 57 °C for the hV1-S1S4. The spectra show that key α-helical secondary structure features are generally retained as a function of temperature, which is consistent with the NMR and fluorescence data that the hV1-S1S4 remains seemingly structured over this temperature range. (**e**) The thermal reversibility of the hV1-S1S4 tested with NMR between 20 °C and 50 °C. Like the reversibility shown with CD in (**c**), the hV1-S1S4 that underwent the cycle of heating and cooling retains the initial resonances as shown in the first 50 °C spectrum (red, left), and the second 50 °C spectrum (dark red, right), showing that this domain does not exhibit thermal hysteresis. Source data are provided as a Source Data file.

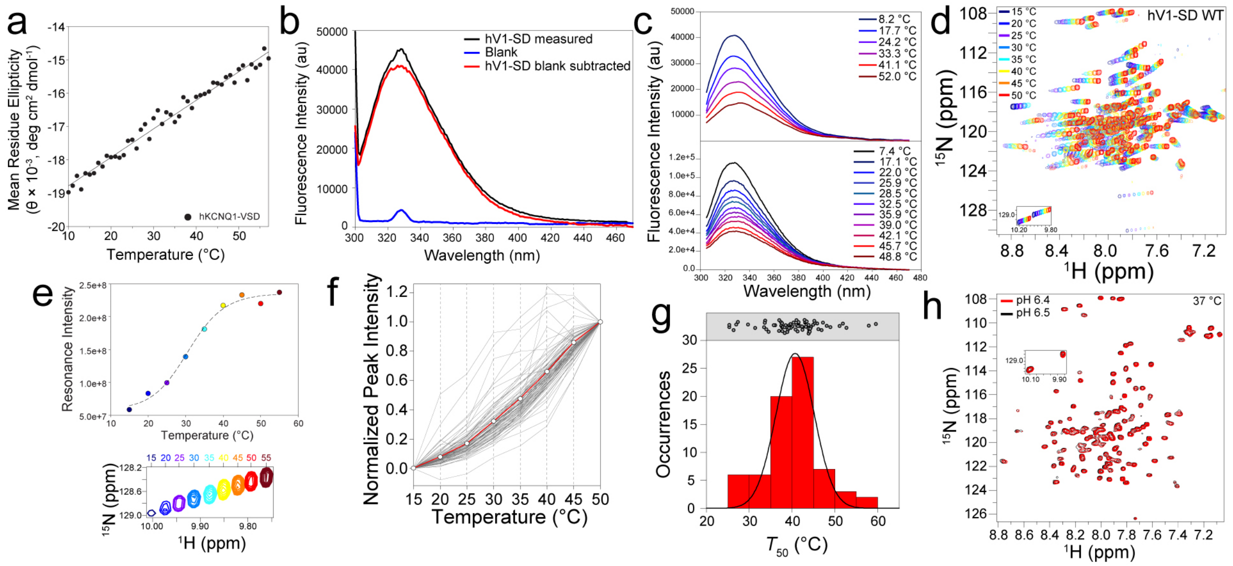

Supplementary Figure 6. Additional data supporting the thermodynamic analyses of the hV1-S1S4 using various biophysical methods. (**a**) Non-temperature sensitive human KCNQ1 voltage-sensing domain was used as a negative control. As expected, the human KCNQ1-VSD shows a linear trend in mean residue ellipticity compared to the hV1-S1S4 data, which shows a clear sigmoidal shape. (**b**) The superimposition of the emission spectra of intrinsic tryptophan fluorescence of the hV1-S1S4, buffer (blank) and the hV1-S1S4 with blank subtracted. (**c**) The superimposition of the emission spectra of the hV1-S1S4 at varying temperature. Two distinct hV1-S1S4 samples were used for these measurements. (**d**) Superimposed ^{1}H-^{15}N TROSY-HSQC spectra of hV1-S1S4 as a function of temperature show significant chemical shift perturbations. The temperature-dependent changes in chemical shift generally reflect the thermal expansion of the hydrogen bonding. The intensity changes as a function of temperature can be explained by the internal dynamics of the individual amide nuclei, which experience linewidth changes that are suitable for thermodynamic analysis. (**e**) Representative NMR data from W549; as the temperature changes, the intensity data has a sigmoidal shape which reflects a two-state transition and can be fit to extract thermodynamic parameters (see Methods). (**f**) Superimposed plots of all assignable NMR resonance intensity against the changes in temperature. The normalized resonance intensities for 71 resonances were plotted as a function of temperature in grey. A resonance that exhibits an apparent two-state model is highlighted in red (G548). (**g**) The histogram distribution of melting temperatures (T_{50}) calculated from the NMR temperature titration with values binned between 25 and 60 °C. This was calculated from fitting the sigmoidal curve. The mean T_{50} of 71 resonances was calculated to be 40.7 ± 0.6 °C. (**h**) The ^{1}H-^{15}N TROSY-HSQC spectra at pH 6.5 and pH 6.4, which is in the range of pH change for the buffer that was used as a function of the temperature ramp, are identical when superimposed. This indicates that temperature induced buffer pH changes are not responsible for the hV1-S1S4 temperature dependent observations noted by NMR and CD experiments. Source data are provided as a Source Data file.

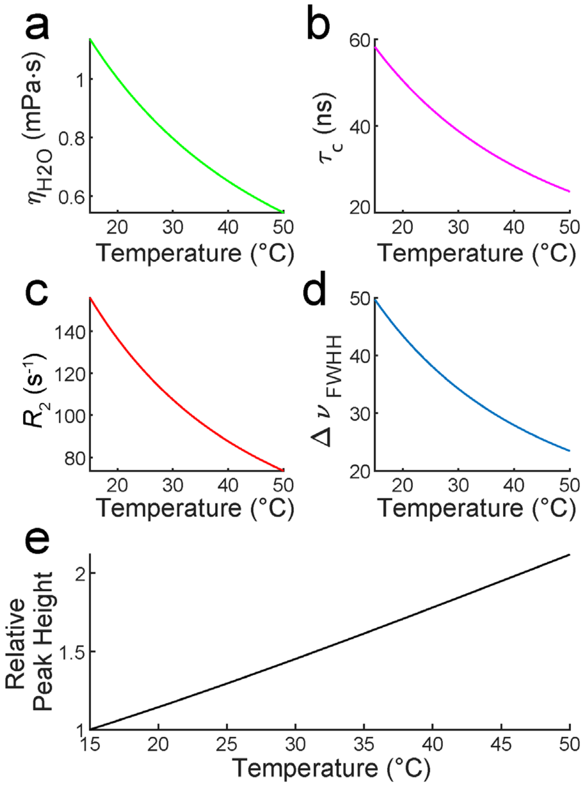

Supplementary Figure 7. Simulated idealized hydrodynamic and NMR parameters for a rigid protein mirroring that of hV1-S1S4. (**a**) The simulated temperature-dependence of the viscosity of water (η_{H_2O}, —) which decreases with increased temperature. (**b**) The correlation time (τ_c, —) similarly decreases with increased temperature. (**c**) The transverse relaxation rate (R_2, —) follows the same trends as the viscosity and correlation time. (**d**) The full peak width at half height ($\Delta\nu_{FWHH}$, —) is proportional to the transverse relaxation rate and has a similar temperature dependence. (**e**) The NMR peak height is inversely proportional to $\Delta\nu_{FWHH}$. Normalizing to the minimum peak height, which in this simulation occurs at 15 °C, shows that there is only a slight non-linearity of the NMR peak height (—) for a protein with the hydrodynamic radius of hV1-S1S4 for data that are recorded at 19.97 T. This apparent compression of the temperature dependence arises from the reciprocal mathematical relationship between peak height and the parameters that underlie its value. Source data are provided as a Source Data file.

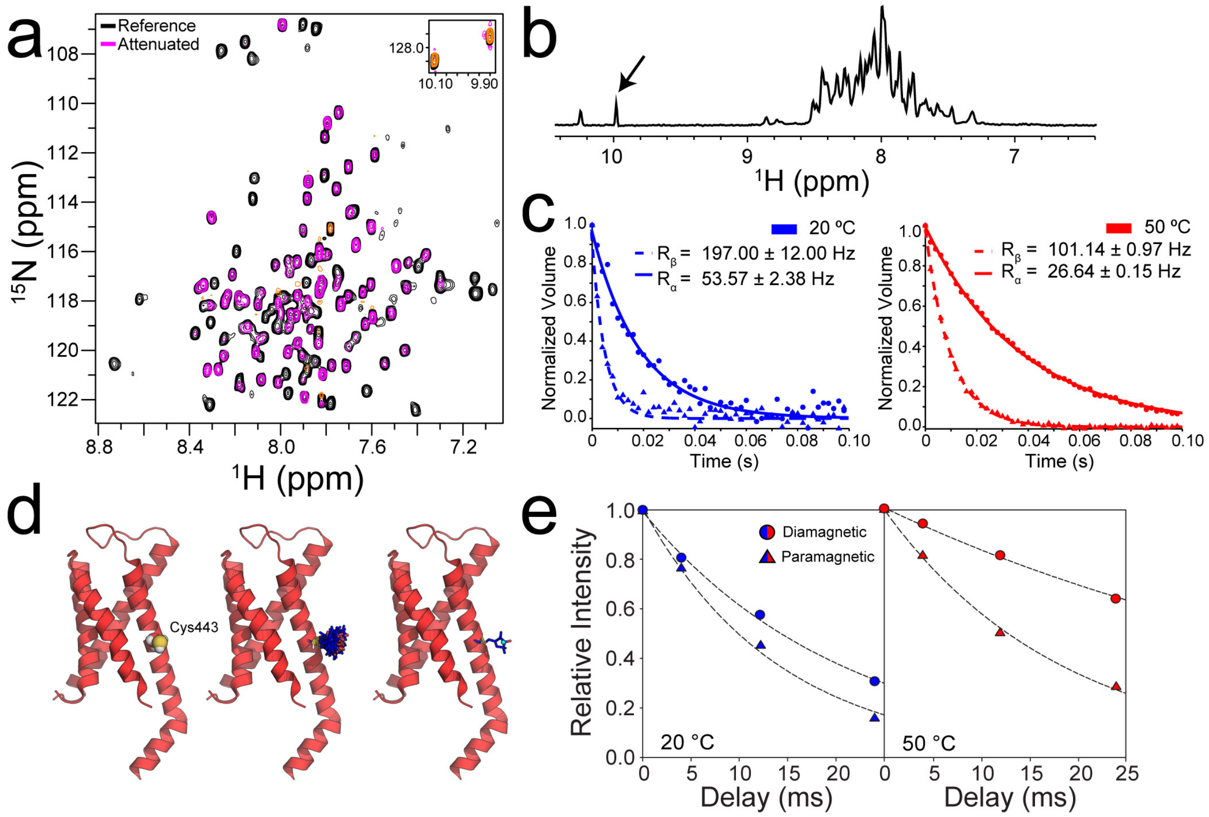

Supplementary Figure 8. Supporting data for RDC and PRE NMR measurements. (a) The reference (black) and attenuated (magenta) ARTSY spectra of the hV1-S1S4 used for the RDC measurements. (**b**) A representative hV1-S1S4 proton NMR spectrum. The indole amine from the S4 helix residue W549, highlighted with an arrow, was used for analysis in the TRACT experiment (see Methods) due to potential artifacts that can arise from dynamic regions in the protein. (**c**) The relaxation rates for TROSY and Anti-TROSY relaxation states from the W549 indole amine at 20 °C and 50 °C. The relaxation rates were calculated by fitting to a monoexponential decay from which the rates were used to calculate the rotational correlation time (τ_c). (**d**) The location of a lone cysteine in the S1 helix of the hV1-S1S4, C443, was labeled with a maleimide paramagnetic nitroxide spin label, MTSL. The middle structure represents the MTSL-labeled S1-S4 domain (rTRPV1. PDB ID: 5IRZ) computationally, with 85 rotameric states of the MTSL modeled in PyMol (see Methods) and right structure displaying the centroid position of all the rotameric states of the MTSL. (**e**) The representative proton relaxation curves for paramagnetic and diamagnetic states at 20 °C and 50 °C for W427. Source data are provided as a Source Data file.

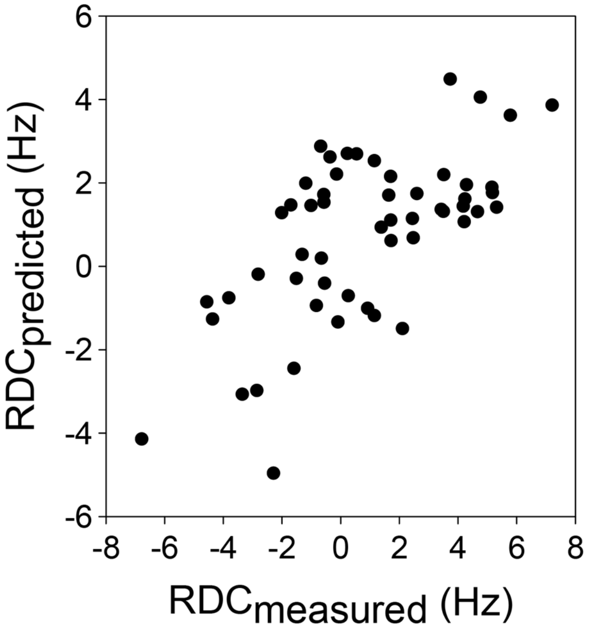

Supplementary Figure 9. Comparison of hV1-S1S4 ^{1}H-^{15}N experimental RDC values with predicted values from the rTRPV1 cryo-EM structure. Experimental ^{1}H-^{15}N RDC values of hV1-S1S4 correlate with RDC values predicted from the cryo-EM structure of apo-rTRPV1 (PDB: 5irz) in nanodiscs using the software PALES. The measured and predicted RDC values correlate with a Pearson's correlation (*R*) of 0.65 and Quality (*Q*) factor of 0.74. The correlation indicates that the general protein fold of the hV1-S1S4 in solution resembles the cryo-EM structure. Source data are provided as a Source Data file.

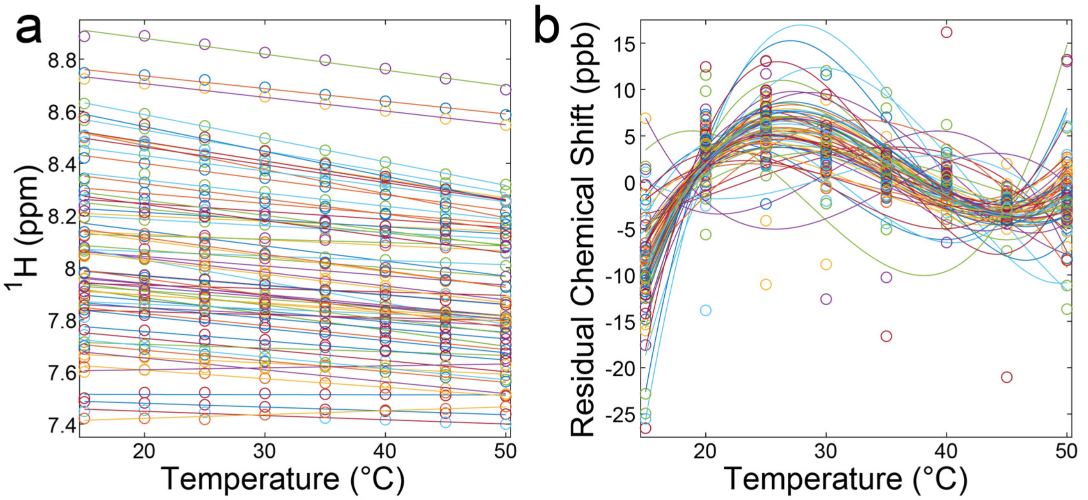

Supplementary Figure 10. Primary amide proton temperature coefficient data and residual chemical shift analysis. (a) Primary data of NMR assigned amide proton resonances (circles) as a function of temperature. Lines are the linear fits of the primary data used to calculate the amide proton temperature coefficients. The average slope, i.e. temperature coefficient, is -4.27 ppb/K. The average mean squared error (MSE) is 0.049 ppb/K. (**b**) Residual chemical shift analysis indicates that the non-linearity of the amide proton temperature dependence is generally quadratic in nature. However, for the hV1-S1S4, the residual chemical shift analysis fits to a cubic polynomial. This is interpreted as arising from temperature dependent conformational change and supporting the temperature-dependent conformational changes detected in NMR peak height, far-UV CD, and intrinsic tryptophan fluorescence experiments. Colors are used only to highlight discrete data series and fits. The rationale is further described in the Methods. Source data are provided as a Source Data file.

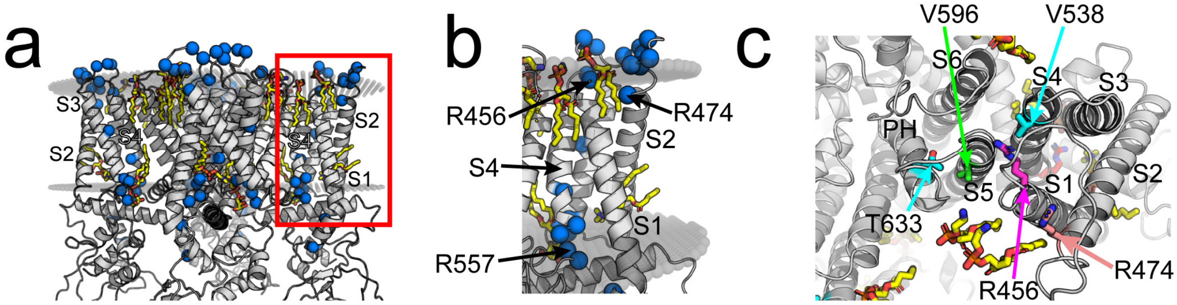

Supplementary Figure 11. Mapping the TRPV1 S1-S4 residues that show temperature-dependent movement. (**a**) The structure of TRPV1 (PDB ID: 5IRZ) in OPM membranes that highlights the residues that show increased solvent accessibility at 45 °C, highlighted with blue. The main chains of the residues with increased solvation are shown in spheres. The S1-S4 domain is displayed in red box. The lipid molecules are shown in yellow sticks. (**b**) A close-up view of the S1-S4 domain. It is clear that the R456 and R557 are embedded in the membrane. All three residues, R456, R474 and R557, have high values of $\Delta NOE_{45°C-20°C}$ suggesting increased solvent exposure at 45 °C and appear to have temperature-dependent movements. (**c**) Extracellular view of the TRPV1 structure (PDB ID: 5IRZ) that highlights key residues in heat and proton activation. R456 (S1 helix, magenta) is known to be crucial in channel function and gating and interacts with V538 (S4 helix, cyan). V538 and T633 (pore helix, cyan) are important for proton activation. V596 (S5 helix, green) is a potential key residue for temperature activation, and this residue is surrounded by R456, V538 and T633.

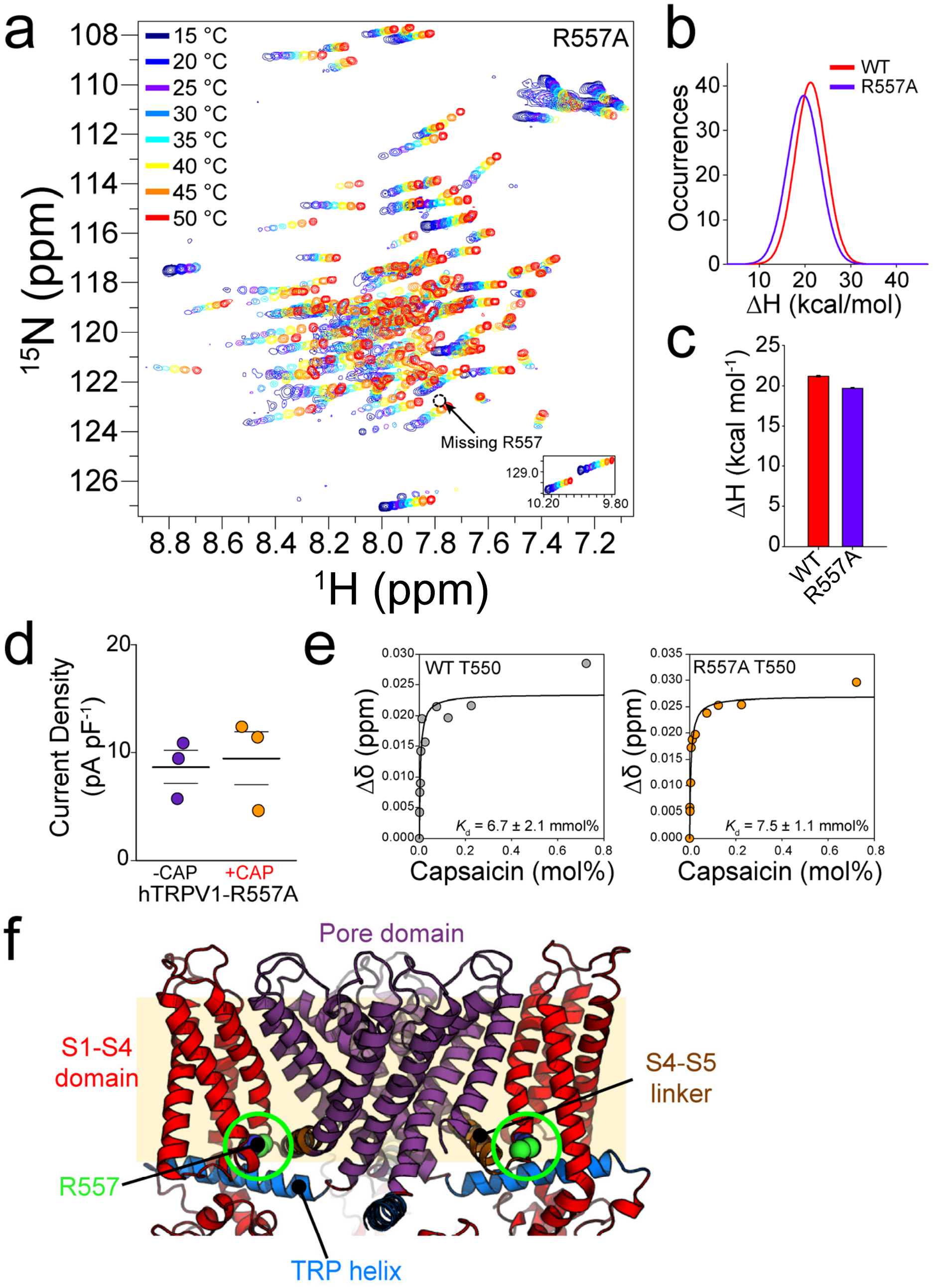

Supplementary Figure 12. Further characterization of R557A hTRPV1 and the R557A hV1-S1S4 domain in temperature and capsaicin activation. (**a**) Superimposed ^{1}H-^{15}N HSQC spectra of the hV1-S1S4-R557A at temperatures from 15 to 50 °C. As expected the R557 resonance is missing in the R557A mutant spectra. (**b**) Comparison in Δ*H* between the WT and the hV1-S1S4-R557A mutant. The Gaussian fittings of the Δ*H* histograms (left) show a leftward shift, indicating that the R557A (purple line) is slightly less temperature sensitive than the WT (red line), consistent with the loss of a cation-π interaction. The ensemble average enthalpy from the respective WT and R557A Gaussian fits and resulting fitting variance are shown in a bar plot (**c**). (**d**) The jittered plot of hTRPV1-R557A of whole-cell patch-clamp electrophysiology in the presence and absence of capsaicin. Consistent with R557A acting as a coupling mutation, the mutant is insensitive to capsaicin activation at a concentration (1 μM) that would saturate WT hTRPV1. Error bars indicate SEM. (**e**) NMR-detected capsaicin binding to the hV1-S1S4 and hV1-S1S4-R557A. T550 in S4 helix is crucial for capsaicin binding, and the R557A mutation does not abrogate the affinity to capsaicin. (**f**) Structure of the rat TRPV1 (PDB ID: 5IRZ) that highlights each structural domain. The S1-S4 domains are highlighted in red. The PD that was implanted into the Shaker is demonstrated in purple. We note that R557 (highlighted in green) is not included in the chimeric study, and the phenotypes of this residue indicate that R557 is central to coupling the S1-S4 domain with the PD in channel gating. Error bars represent SEM. Source data are provided as a Source Data file.

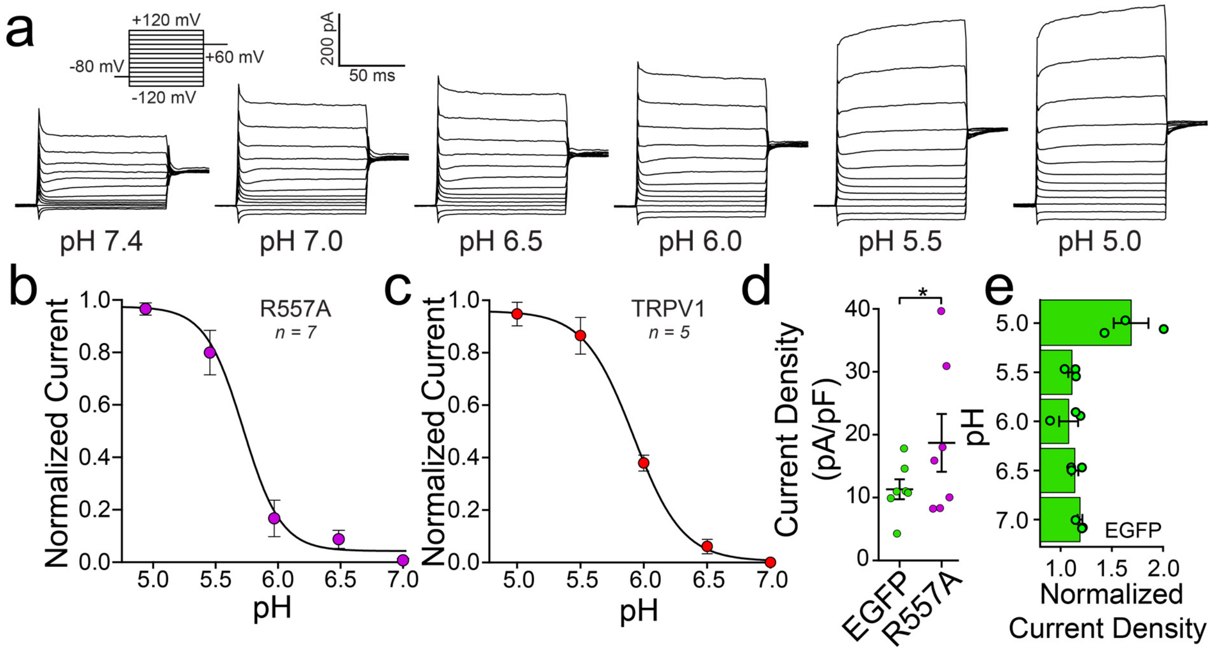

Supplementary Figure 13. hTRPV1 R557A is a functional proton activated channel. (**a**) Whole-cell current traces from the average of seven cells expressing R557A hTRPV1 incrementally exposed to decreasing pH values show that the mutant channel is activated by protons. Inset is the voltage step protocol used for pH measurements. (**b**) pH-response curve for R557A hTRPV1 shows a pH sensitivity that is similar to WT hTRPV1 (**c**). R557A data are shown as the normalized mean ± SEM (n = 7) measured at +100 mV. Fitting the R557A data (purple) to a modified Hill equation gives an EC_{50} = 5.72 ± 0.06 with a Hill coefficient of -2.8 ± 0.7. (**c**) The equivalent proton activation studies of wild-type hTRPV1 (red, n = 5) identify an EC_{50} = 5.92 ± 0.03 with a Hill coefficient of -2.2 ± 0.2. (**d**) Comparison of R557A (purple) currents elicited at +100 mV and pH 7.4 with cells that were transfected with empty pIRES2-EGFP vector (EGFP, green) at the same conditions show that the R557A increases basal current density suggestive of R557A-hTRPV1 trafficking and channel function. The mean ± SEM for n = 7 cells for each condition results in a P = 0.0401 from an unpaired students T-test. (**e**) pH characterization of HEK293 cells transfected with empty vector (pIRES2-EGFP, n = 3) shows that there is a minimal pH response in the absence of R557A-hTRPV1. Taken together these data support that R557A-hTRPV1 is a functional channel that is S1-S4 coupling defective. Error bars represent SEM. Source data are provided as a Source Data file.

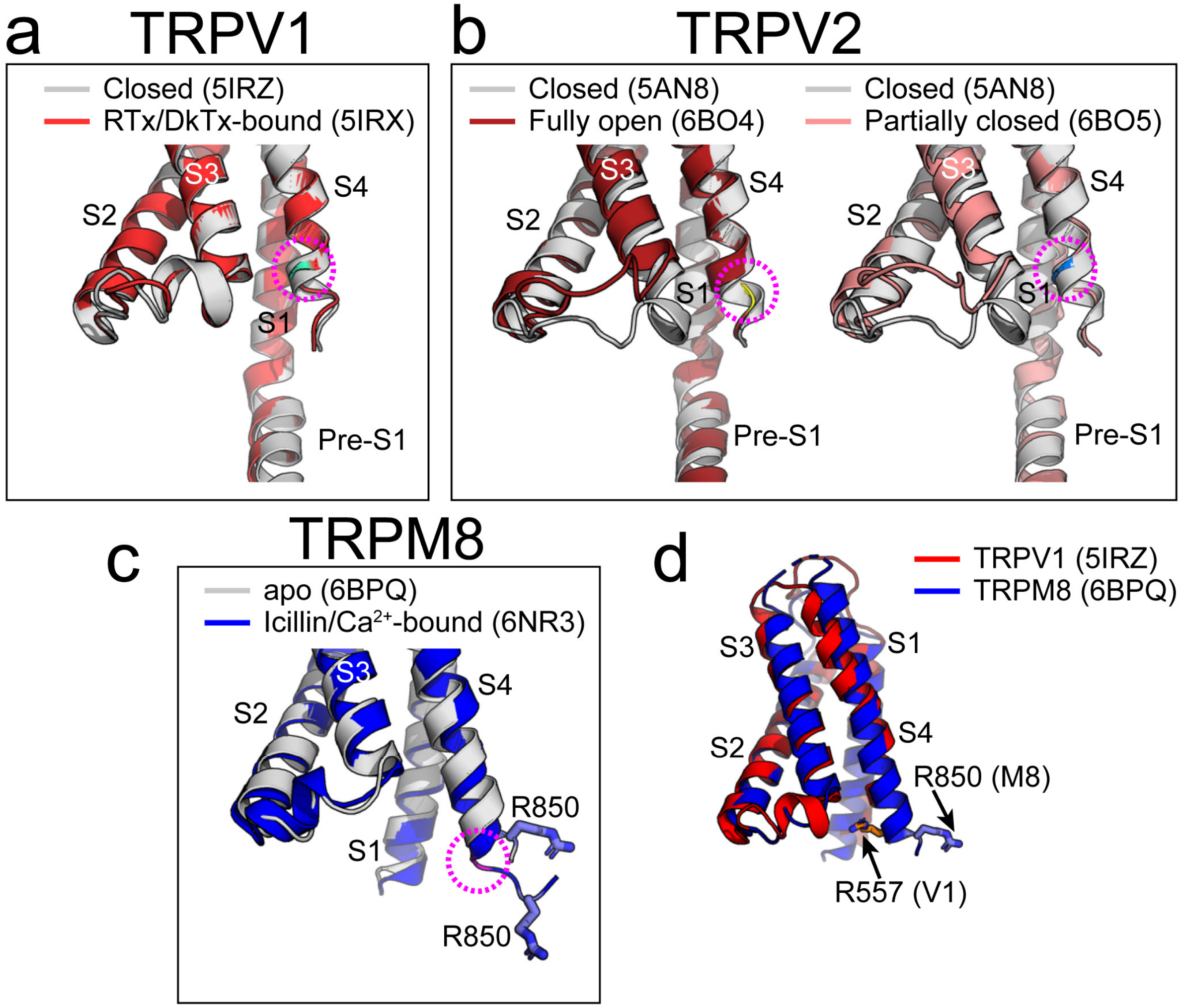

Supplementary Figure 14. Structural examples of S4 helix motions in TRP channel activation. (**a**) Superimposed structures of TRPV1 in its resting (PDB ID: 5IRZ, light grey) and active states (RTx/DkTx-bound, PDB ID: 5IRX, red). In the active state, starting around Y555 (light blue, highlighted with magenta circle), the bottom of the S4 helix starts unfurling. (**b**) The same unfurling trend found in TRPV2. The fully-closed structure of TRPV2 (PDB ID: 5AN8) is superimposed with the fully open state that includes the pore turret (PDB ID: 6BO4, dark red on the left), and partially closed state which does not include the pore turret (PDB ID: 6BO5, pink on the right). In both alignments, the unfurling of the S4 begins at the bottom of the S4 helix (highlighted in yellow and blue, with magenta circle). (**c**) Superimposed structures of the apo-TRPM8 (PDB ID: 6BPQ, light grey), and the icilin/Ca^{2+}-bound TRPM8 (PDB ID: 6NR3, blue). In TRPM8, the bottom of the S4 helix undergoes an extreme conformational change, induced by the α- to 3_{10}- helix transition, starting at the residue highlighted in magenta. This difference can be verified by the displacement of R850, as it travels further down the membrane when TRPM8 is activated. (**d**) The alignment of the S1-S4 domains of TRPV1 (red) and TRPM8 (blue). Although the orientation is different, R557 in TRPV1 and R850 in TRPM8 are located in the C-terminal end of the S4 helix, near the membrane bilayer.

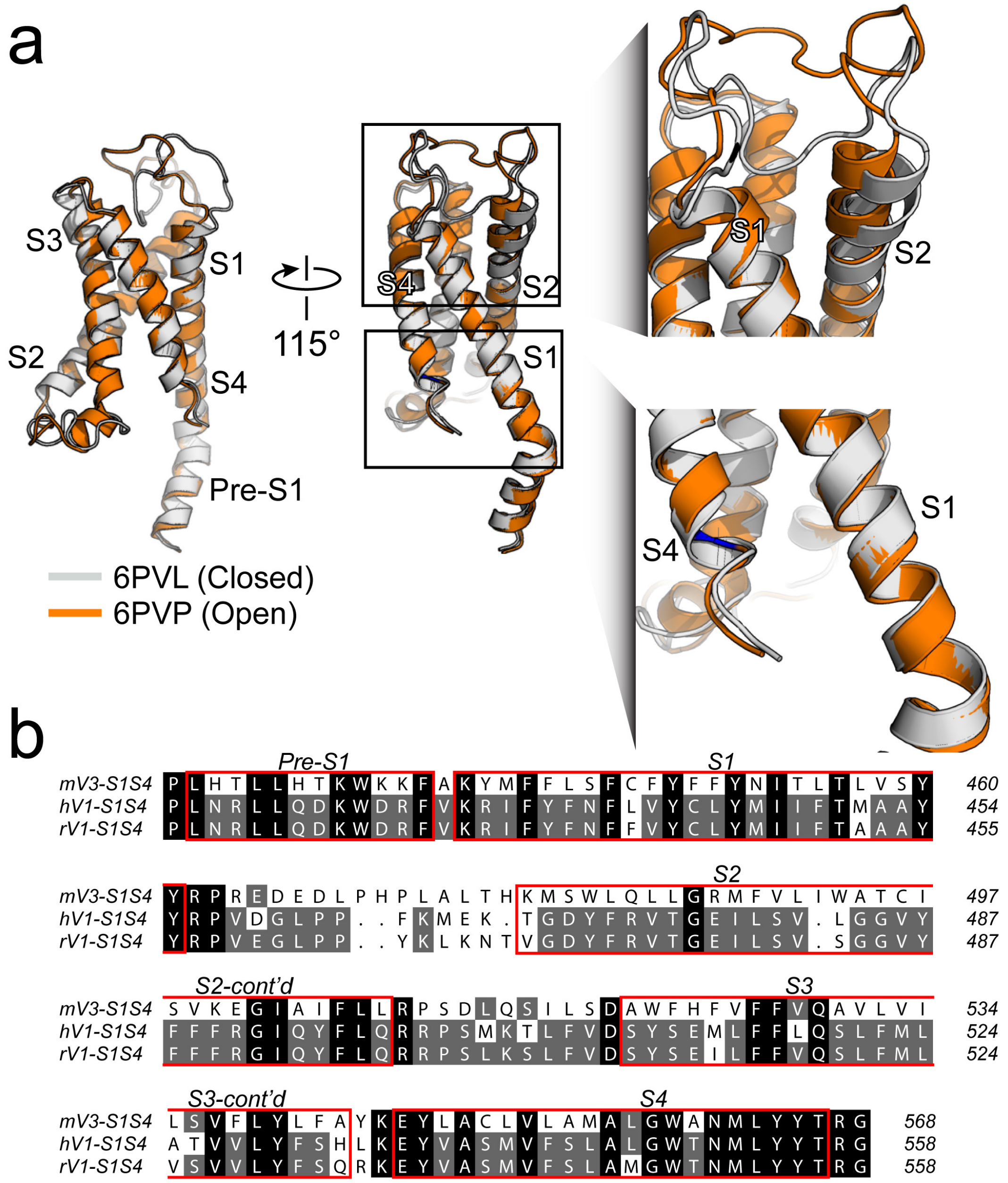

Supplementary Figure 15. Putative temperature-dependent motions in the TRPV3 S1-S4 domain. (**a**) Superimposed structures of mouse TRPV3 in its closed state (PDB ID: 6PVL, light grey, wild-type channel at 42 °C) and temperature activated state (PDB ID: 6PVP, orange, Y564A mutant channel at 37 °C). As highlighted in the lower right of panel (a), DSSP analysis of the pdb structures of the closed and temperature-activated states of the S4 helices indicate a change in helical character. In the closed state structure (6PVL), the C-terminus of the S4 helix, transitions from α-helix to 3_{10}-helix at residue A560 and from 3_{10}-helix to loop at T566. However, the active state secondary structure (6PVP) as determined by DSSP, indicates that there is no 3_{10}-helix and the α-helix transition to loop begins after M562. The upper right panel in A, identifies that the loop between S1 and S2 appears to be membrane-associated in the closed state and solvent-exposed in the temperature-active state. This loop conformational change appears to impact the extracellular side of the S1, S2, and S3 transmembrane helices. Both of these TRPV3 structural changes are consistent with temperature-dependent motions of the TRPV1 S1-S4 identified in the current study. (**b**) Alignment of the mouse TRPV3, rat TRPV1, and human TRPV1 S1-S4 domain sequences.

Supplementary Tables

Supplementary Table 1. Measured thermosensitivity values (ΔH*)* from TRPV1 temperature studies.

ΔH (kcal/mol)	Potential (mV)	Method	TRPV1 species	Protein Origin/ Membrane Type	Reference
98 ± 12	+60	Whole-cell	Human	HEK293/HEK293	**This Study (Steady state)**
94 ± 8	+60	Whole-cell	Human	HEK293/HEK293	**This Study (Temp ramp)**
64.9[a]	-70	Whole-cell	Rat	HEK293/HEK293	**Vlachová et al., 2003**
150 ± 13	-60	Inside-out	Rat	*X. laevis/ X. laevis*	**Liu et al., 2003**
26.8	+80	Inside-out	Murine	HEK293/HEK293	**Yang et al., 2010**
101 ± 4<br>65 ± 6	-60, +60	Outside-out	Rat	HEK293/HEK293	**Yao et al., 2010**
90 ± 3	-60	Whole-cell	Rat	HEK293/HEK293	**Yao et al., 2011**
86.2 ± 3.9[b]	-60	Proteoliposome patch	Rat	Sf9 Insect cells/Soybean polar lipids	**Cao et al., 2013**
155	+100	Single-channel planar lipid bilayers	Rat	HEK293/3:1 POPC:POPE	**Sun and Zakharian, 2015**
65 ± 5.7[a]	-60	Whole-cell	Rat	*X. laevis/ X. laevis*	**Zhang et al., 2017**
88 ± 8	+60	Inside-out	Rat	HEK293/HEK293	**Sánchez-Moreno et al., 2018**

[a]*The reported temperature coefficient (*Q_{10}*) was converted to ΔH according to* $\Delta H \approx 20lnQ_{10}$ [5].
[b] *Protein was purified and reconstituted in proteoliposomes.*

Supplementary Table 2. Buffer pH stability as a function of temperature.

Temperature (°C)[a]	pH[b]
10	6.49
15	6.50
20	6.49
25	6.48
30	6.46
35	6.41
40	6.41
45	6.38
50	6.36
55	6.40
60	6.39

[a] *Temperature measured with Fluke 52 II thermometer with 80PK-1 beaded type-K thermocouple; according to the instrument specifications, in this temperature range, should produce a maximum temperature error of ±0.4 °C.*
[b] *This data is for the phosphate buffer used in CD, fluorescence, and NMR measurements (see Methods).*

Supplementary Table 3. Comparison between ^{1}H-^{15}N RDCs of hV1-S1S4 and values predicted from cryo-EM structures of TRPV1 and TRPV3.

Protein	***PDB***	***Res (Å)***	***State***	***Corr R***	***Q factor***	***RMS (Hz)***
rTRPV1	5irz	3.28	Apo	0.646	0.735	2.294
	5is0*	3.43	Antagonist-bound	0.630	0.748	2.330
	5irx†	2.95	Agonist-bound	0.512	0.830	2.589
mTRPV3	6pvl	4.4	42 °C/Closed	0.570	0.795	2.384
	6pvp‡	4.48	37 °C/Open	0.415	0.881	2.642

**Complex with capsazepine (CPZ)*

† Complex with resiniferatoxin (RTX) and the tarantula double knot toxin (DkTx)

‡ Structure is of the TRPV3 mutant Y564A. mTRPV3 Y564 is equivalent to Y554 in hTRPV1.

Supplementary Table 4. TRPV1 DNA primers.

Primer Name	Sequence
Mutagenic Primers	
hTRPV1_QC_R557A_FW†	CCTTGGGCTGGACCAACATGCTCTACTACACCGCCGGTTT
hTRPV1_QC_R557A_RV†	AAACCGGCGGTGTAGTAGAGCATGTTGGTCCAGCCCAAGG
hV1-S1D4_R557A_FW‡	TGCTGTATTACACCGCTGGTTAAGGATCC
hV1-S1D4_R557A_RV‡	GCCGGATCCTTAACCAGCGGTGTAATACAG
Sequencing Primers	
pIRES-Seq-FW†	AATGGGCGGTAGGCGTGTA
pIRES-Seq-RV†	TCACATTGCCAAAAGACGG
hV1_Int_FW1†	GAAGACCTGTCTGCTGAAAG
hV1_Int_FW2†	CTTGGCCTATATTCTCCAG
hV1_Int_FW3†	ACCAACATGCTCTACTACAC
T7-FW‡	TAATACGACTCACTATAGGG
T7-RV‡	GCTAGTTATTGCTCAGCGG
Cloning Primers	
hTRPV1_BamHI_RV†*	ATCAGACGGATCCTCACTTCTCCCCGGAAGC
hTRPV1_SalI_FW†*	GCGGTACGTCGACATGAAGAAATGGAGCAGCACAGACTTG

†Primer used with the full-length hTRPV1
‡ Primer used with the hV1-S1S4
*hTRPV1 was amplified from a human TRPV1 cDNA cloning vector integrated in a PCR-XL-TOPO vector backbone

Supplementary Table 5. Parameters used to simulate the temperature dependence of NMR peak height.

Parameter/Constant	Value
^{1}H frequency*	$\omega_H = 850.279 \times 10^6\ Hz$
^{15}N frequency*	$\omega_N = 86.168 \times 10^6\ Hz$
^{1}H gyromagnetic ratio	$\gamma_H = 2.6752 \times 10^8\ T^{-1} \cdot s^{-1}$
^{15}N gyromagnetic ratio	$\gamma_N = -2.713 \times 10^7\ T^{-1} \cdot s^{-1}$
Reduced Planck constant	$\hbar = 1.0546 \times 10^{-34} J \cdot s$
Vacuum permeability	$\mu_0 = 4\pi \times 10^{-7}\ T^2 \cdot m^3 \cdot A^{-1}$
Amide bond length†	$r_{HN} = 1.023$ Å
^{15}N chemical shift anisotropy‡	$\Delta\sigma = -171.7\ ppm$
hV1-S1S4 Stokes radius	$r_{H-hV1S1S4} = 36.5$ Å

*Frequencies at 19.97 T, that used in the reported NMR experiments.

†HN amide bond distance, r_{HN} was taken from Yao et al.[18]

‡This is an average value from a 16.7 kDa membrane-bound protein[19] which agrees with that determined from a soluble protein (-172 ppm)[20]

Supplementary References

1. Cavanagh, J.F., W. J.; Palmer III, A. G.; Rance, M.; Skelton, N. J. *Protein NMR Spectroscopy: Principles and Practice (Second Edition)*, (Elsevier Academic Press., 2007).
2. Greenfield, N.J. Using circular dichroism collected as a function of temperature to determine the thermodynamics of protein unfolding and binding interactions. *Nat. Protoc.* **1**, 2527-35 (2006).
3. Privalov, P.L. & Potekhin, S.A. Scanning microcalorimetry in studying temperature-induced changes in proteins. *Methods Enzymol.* **131**, 4-51 (1986).
4. Voets, T. et al. The principle of temperature-dependent gating in cold- and heat-sensitive TRP channels. *Nature* **430**, 748-54 (2004).
5. Clapham, D.E. & Miller, C. A thermodynamic framework for understanding temperature sensing by transient receptor potential (TRP) channels. *Proc. Natl. Acad. Sci. USA* **108**, 19492-7 (2011).
6. Chowdhury, S., Jarecki, B.W. & Chanda, B. A molecular framework for temperature-dependent gating of ion channels. *Cell* **158**, 1148-58 (2014).
7. Prabhu, N.V. & Sharp, K.A. Heat capacity in proteins. *Annu. Rev. Phys. Chem.* **56**, 521-48 (2005).
8. Voets, T. TRP channels and thermosensation. *Handb. Exp. Pharmacol.* **223**, 729-41 (2014).
9. Yang, F., Cui, Y., Wang, K. & Zheng, J. Thermosensitive TRP channel pore turret is part of the temperature activation pathway. *Proc. Natl Acad. Sci. USA* **107**, 7083-8 (2010).
10. Yao, J., Liu, B. & Qin, F. Kinetic and energetic analysis of thermally activated TRPV1 channels. *Biophys. J.* **99**, 1743-53 (2010).
11. Yao, J., Liu, B. & Qin, F. Modular thermal sensors in temperature-gated transient receptor potential (TRP) channels. *Proc. Natl Acad. Sci. USA* **108**, 11109-14 (2011).
12. Voets, T., Owsianik, G., Janssens, A., Talavera, K. & Nilius, B. TRPM8 voltage sensor mutants reveal a mechanism for integrating thermal and chemical stimuli. *Nat. Chem. Biol.* **3**, 174-82 (2007).
13. Korson, L., Drost-Hansen, W. & Millero, F.J. Viscosity of water at various temperatures. *The Journal of Physical Chemistry* **73**, 34-39 (1969).
14. Kay, L.E., Torchia, D.A. & Bax, A. Backbone dynamics of proteins as studied by 15N inverse detected heteronuclear NMR spectroscopy: application to staphylococcal nuclease. *Biochemistry* **28**, 8972-9 (1989).
15. Lipari, G. & Szabo, A. Model-free approach to the interpretation of nuclear magnetic resonance relaxation in macromolecules. 1. Theory and range of validity. *Journal of the American Chemical Society* **104**, 4546-4559 (1982).
16. Cao, X., Ma, L., Yang, F., Wang, K. & Zheng, J. Divalent cations potentiate TRPV1 channel by lowering the heat activation threshold. *J Gen Physiol* **143**, 75-90 (2014).
17. Yang, F., Ma, L., Cao, X., Wang, K. & Zheng, J. Divalent cations activate TRPV1 through promoting conformational change of the extracellular region. *J Gen Physiol* **143**, 91-103 (2014).
18. Yao, L., Vogeli, B., Ying, J. & Bax, A. NMR determination of amide N-H equilibrium bond length from concerted dipolar coupling measurements. *J Am Chem Soc* **130**, 16518-20 (2008).
19. Pandey, M.K. et al. Determination of 15N chemical shift anisotropy from a membrane-bound protein by NMR spectroscopy. *J Phys Chem B* **116**, 7181-9 (2012).
20. Kroenke, C.D., Rance, M. & Palmer, A.G. Variability of the15N Chemical Shift Anisotropy inEscherichia coliRibonuclease H in Solution. *Journal of the American Chemical Society* **121**, 10119-10125 (1999).
