# Supplementary material for: Evidence that the TRPV1 S1-S4 membrane domain contributes to thermosensing
Source: Nat Commun. 2020 Aug 20;11:4169. doi: 10.1038/s41467-020-18026-2 (PMC7441067; doi:10.1038/s41467-020-18026-2)
Supplement: Supplementary file 5 — Additional Source Data [file 41467_2020_18026_MOESM5_ESM.zip › Nat_Commun_Source_Data_Final/FigS1/panel b/Arizona State University MSB-3799 081114.pdf]

MS Bioworks
Protein Mass Spectrometry Services

3950 Varsity Drive
Ann Arbor, MI 48108
734-929-5083
www.msbioworks.com
info@msbioworks.com

Project Report

Information

Client:	Wade Van Horn
Institute:	Arizona State University
Project Number:	MSB-3799
Date Submitted:	August 5th, 2014
Date Completed:	August 11th, 2014

Samples

Client identifier	MSB identifier
hPIRT	18220
hTRPV1-VSD	18221

Objective

Identify the protein content of two submitted gel bands using in-gel digestion, LC/MS/MS and database searching.

Experimental Methods

Sample Preparation

Trypsin digestion was performed using a robot (ProGest, DigiLab) with the following protocol:

Washed with 25mM ammonium bicarbonate followed by acetonitrile.
Reduced with 10mM dithiothreitol at 60°C followed by alkylation with 50mM iodoacetamide at RT.
Digested with trypsin (Promega) at 37°C for 4h.
Quenched with formic acid and the supernatant was analyzed directly without further processing.

Mass Spectrometry

The gel digest was analyzed by nano LC/MS/MS with a Waters NanoAcquity HPLC system interfaced to a ThermoFisher Q Exactive. Peptides were loaded on a trapping column and eluted over a 75µm analytical

column at 350nL/min; both columns were packed with Jupiter Proteo resin (Phenomenex). The mass spectrometer was operated in data-dependent mode, with MS and MS/MS performed in the Orbitrap at 70,000 FWHM resolution and 17,500 FWHM resolution, respectively. The fifteen most abundant ions were selected for MS/MS.

Data Processing

Data were searched using a local copy of Mascot with the following parameters:

Enzyme: Trypsin
Database: Uniprot E. coli (forward and reverse appended with common contaminants and hPIRT and hTRPV1-VSD sequences)
Fixed modification: Carbamidomethyl (C)
Variable modifications: Oxidation (M), Acetyl (Protein N-term), Pyro-Glu (N-term Q), Deamidation (NQ)
Mass values: Monoisotopic
Peptide Mass Tolerance: 10 ppm
Fragment Mass Tolerance: 0.02 Da
Max Missed Cleavages: 2

Mascot DAT files were parsed into the Scaffold software for validation, filtering and to create a non-redundant list per sample. Data were filtered using a minimum protein value of 90%, a minimum peptide value of 50% (Prophet scores) and requiring at least two unique peptides per protein.

Results

The Scaffold file for this study contains all search results, coverage maps, peptide lists and product ion data. This will allow you to visualize much more than we can describe in this report. You may download your Scaffold file from:

ftp://

Username:
Password:

A total of 15 proteins were detected with two or more unique peptides across both samples. Please refer to the accompanying Excel and Scaffold files. A brief overview of protein and peptide totals is shown here:

MSB Identifier	18220	18221
Total No. of Proteins	13	9
Total No. of Spectra Matching	232	193
Total No. of Unique Peptides	84	59

The target proteins were correctly identified as the most abundant proteins in both samples:

18220 (hPIRT)

hPIRT (100%), 16,403.1 Da
hPIRT
19 exclusive unique peptides, 42 exclusive unique spectra, 138 total spectra, 91/146 amino acids (62% coverage)

```
MGHHHHHHGM TMETLPKVLE VDEKSPEAKD LLPSQTASSL CISSRSESVW
TTTPRSNWEI YRKPIVIMSV GGAILLFGVV ITCLAYTLKL SDKSLSILKM
VGPGFLSLGL MMLVCGLVWV PIIKKKQKHR QKSNFLRSLK SFFLTR
```

18221 (hTRPV1-VSD)

hTRPV1-VSD (100%), 19,103.1 Da
hTRPV1-VSD
18 exclusive unique peptides, 53 exclusive unique spectra, 139 total spectra, 81/159 amino acids (51% coverage)

```
MGHHHHHHHH HHLVPRGSPL NRLLQDKWDR FVKRIFYFNF LVYCLYMIIF
TMAAYYRPVD GLPPFKMEKT GDYFRVTGEI LSVLGGVYFF FRGIQYFLQR
RPSMKTLFVD SYSEMLFFLQ SLFMLATVVL YFSHLKEYVA SMVFSLALGW
TNMLYYTRG
```
