# Supplementary figures and images for: Evidence that the TRPV1 S1-S4 membrane domain contributes to thermosensing
Source: Nat Commun. 2020 Aug 20;11:4169. doi: 10.1038/s41467-020-18026-2 (PMC7441067; doi:10.1038/s41467-020-18026-2)

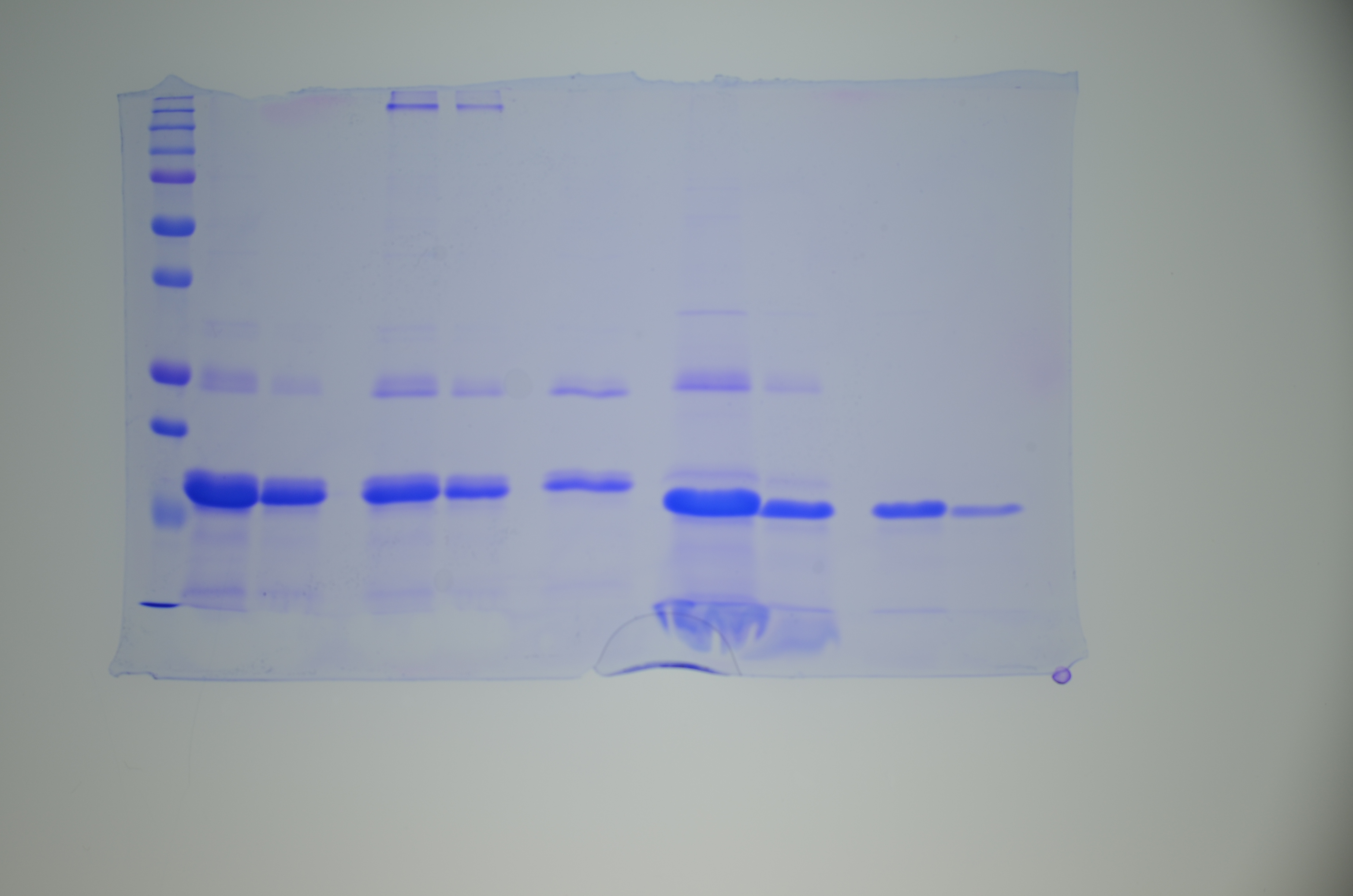

Supplement: Supplementary file 5 — Additional Source Data [file 41467_2020_18026_MOESM5_ESM.zip › Nat_Commun_Source_Data_Final/FigS1/panel a/hV1-S1S4_SDS-PAGE_full_gel.jpg]

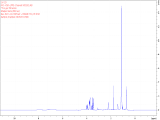

Supplement: Supplementary file 5 — Additional Source Data [file 41467_2020_18026_MOESM5_ESM.zip › Nat_Commun_Source_Data_Final/FigS8/panel b/FigS8b_hV1-S1S4_1D/pdata/1/thumb.png]
