# Supplementary material for: Evidence that the TRPV1 S1-S4 membrane domain contributes to thermosensing
Source: Nat Commun. 2020 Aug 20;11:4169. doi: 10.1038/s41467-020-18026-2 (PMC7441067; doi:10.1038/s41467-020-18026-2)
Supplement: Supplementary file 2 — Peer Review File [file 41467_2020_18026_MOESM2_ESM.docx]

Peer Review Information

**Journal:** Nature Communications

**Manuscript title:** Evidence that the TRPV1 S1-S4 Membrane Domain Contributes to Thermosensing

**Corresponding author name(s):** Prof Wade Van Horne

**Editorial notes:**

**Transferred manuscripts**

This manuscript has been previously reviewed at another journal that is not operating a transparent peer review scheme. This document only contains reviewer comments and rebuttal letters for versions considered at *Nature Communications*.

**Transferred manuscripts (no peer review at *Nature Communications*)**

This manuscript has been previously reviewed at another journal that is not operating a transparent peer review scheme. The manuscript was considered suitable for publication without further review at *Nature Communications*.

**Redactions – transferred manuscripts (mention of previous referee reports from elsewhere)**

This manuscript has been previously reviewed at another journal that is not operating a transparent peer review scheme. This document only contains reviewer comments and rebuttal letters for versions considered at *Nature Communications*. Mentions of prior referee reports have been redacted.

**Redactions – transferred manuscripts (mention of the other journal)**

This manuscript has been previously reviewed at another journal that is not operating a transparent peer review scheme. This document only contains reviewer comments and rebuttal letters for versions considered at *Nature Communications*. Mentions of the other journal have been redacted.

**Redactions – unpublished data**

Parts of this Peer Review File have been redacted as indicated to maintain the confidentiality of unpublished data.

**Redactions – confidential patient information**

Parts of this Peer Review File have been redacted as indicated to maintain patient confidentiality.

**Redactions – published data**

Parts of this Peer Review File have been redacted as indicated to remove third-party material where no permission to publish could be obtained.

**Amendments – published data**

[Figure X/The figure on page X] in this Peer Review File has been amended to remove third-party material where no permission to publish could be obtained.

**Published data (credit line for image)**

[Figure X/The figure on page X] in this Peer Review File is reproduced with permission from [Copyright Holder].

**Redactions – reviewer opt-out**

Parts of this Peer Review File have been redacted as indicated as we could not obtain permission to publish the reports of reviewer ###.

**Reviewer comments in marked-up manuscript**

In their review of the [first/second/third/...] version of this manuscript, reviewer ### added their comments to the manuscript file. These comments, excluding minor textual revisions, have been copied into this Peer Review File.

**Reviewer comments & decisions:**

**Reviewer comments, first version:**

Reviewers' comments:

Reviewer #1 (Remarks to the Author):

In this manuscript, Kim et al. use biophysical methods (solution NMR, Far-UV CD, and intrinsic fluorescence) to study temperature-dependent conformational changes in the S1-S4 domain of the heat-activated cation channel TRPV1. They find that this isolated region undergoes a temperature-dependent transition that is consistent with the temperature dependence of gating in the intact channel.
Overall, this is an interesting study that provides molecular and biophysical details regarding the structural changes in a functionally important region of the channel. However, the conclusions that can be drawn from this study regarding the gating of the intact channel are limited, as direct evidence that the observed conformational change is actually coupled to opening of the channel is lacking. The functional patch-clamp data have their limitations, and do not allow strong conclusions.

Specific points
1) The authors use the TRPV1-R557A mutation, claiming that the mutated residue is essential for coupling the S1-S4 domain to the gate. Then, the authors show that this mutant cannot be activated by heat or capsaicin, and attribute this to the deficient coupling. However, there is no evidence that the mutated channel is active at all - both tested stimuli are negative, and the currents shown in Figure 4a do not look at all like TRPV1-mediated currents. In the absence of any evidence that the mutant channel is correctly expressed in the membrane and can be activated, these results are meaningless.

2) The authors use a K-based pipette solution (I suppose that 315 mM K+-gluconate is a typo). As a result, a significant part of the outward currents in HEK cells will be carried by endogenous K-channels, which explains the characteristics of the outward currents in Figure 4a.

3) To address points 1 and 2, I suggest the authors perform experiments with a Cs-or Na-based pipette solution, and evaluate whether the mutant channel can be activated by other stimuli such as low pH.

4) As indicated by the authors, also other parts of TRPV1 have been implicated in heat sensing in other studies (basically every region of the channel has been proposed). Therefore, one could conclude that the temperature-dependent transitions are not localised to a specific region (e.g. S1-S4), but occur allover the channel. If the authors want to propose that S1-S4 is somehow of special importance, further evidence is needed to show that specific mutations in this region, affecting the described structural transitions, affect heat-induced channel gating in a consistent manner.


Reviewer #2 (Remarks to the Author):

Kim and Sisco et al investigated the isolated TRPV1 ion channel S1-S4 transmembrane bundle and its response to temperature and capsaicin. This is a highly relevant question as TRPV1 is one of the best studied protein thermosensors, with an activation temperature of ~42C, yet the molecular details of thermosensing remain unclear. Previous work has suggested that the residues and/or region(s) required for thermosensing are localized in the transmembrane region.
The authors took advantage of the fact that the isolated TRPV1 S1-S4 transmembrane domain shows clear evolutionary relationships with the sensing domains of other ion channels. They purified the isolated human TRPV1 S1/S4 domain and, using a combination of solution NMR, CD and fluorescence spectroscopy, investigate its interaction with capsaicin and its conformational response to temperature. Notably, the authors present NMR spectra of this transmembrane domain in LPPG micelles which are of exceptional quality, thus allowing them to confidently assign the majority of the backbone resonances and to derive a number of other informative NMR parameters.
They present the first convincing data that the S1-S4 bundle undergoes well-defined local structural changes in solution in response to temperature that suggest a putative molecular mechanism for temperature sensing in this class of ion channels. These are highly interesting results and of broad relevance to a large number of researchers and therefore this manuscript is in general well suited for publication in Nature Communications if the authors address a few technical points:

a) Fig. 1b: Can the color code be changed, the apo state spectra (black) are almost impossible to see.

b) Fig. 1c: Can the authors please clarify their color code (light/dark grey) in the figure legend for better readability?

c) Capsaicin interaction: The authors observe that the residues previously identified to be relevant for capsaicin binding also interact with capsaicin in the context of the isolated S1/S4 bundle, but from the HSQC in Fig. 1d its apparent that a large number of peaks are affected by capsaicin, thsu suggesting that in addition to the residues named by the authors, also neighboring amino acids or even allosteric sites are affected. NMR is a particularly valuable technique to identify such effects. I therefore strongly recommend to plot the chemical shift pertubation on a structural model of the S1/S4 bundle. This will help the reader and may teach us something about the long(er) range effects of capsaicin binding.

d) The temperature sensing experiments are very exciting. van Horn and colleagues present an elegant combination of electrophysiology with CD, Trp fluorescence and NMR spectroscopy to measure the temperature dependent transition between two states. Could the authors please also specifically mention the inflection point temperatures for ALL the different experimental approaches (also for CD and fluorescence) in the figure legend so that these differences can be discussed? It would help readability if these values were all presented in one place.

e) In Fig. 2d, the conformational changes related to temperature sensing are demonstrated to manifest themselves in the sigmoidal character of the peak intensity vs temperature curve. This is only shown for a single residue in Fig 2d and another one in the supplements. It would be nice to see more examples included in the supplementary materials.
Are there residues far away from the residues affected by heat-induced conformational changes that do not display a sigmoidal curve? These should be shown for comparison.

f) Fig 2d: It is unclear what signal intensity refers to in this graph – is this signal height or the integral intensity? This needs to be clarified. In case it is the signal height, the authors should show a simulated graph of peak heights vs temperature for a rigid protein of the same size and correlation time since the linewidth for such a system also changes as a function of temperature and correlation times.

g) Dividing the DH values (in Fig 2f) by 4 to derive the contributions of the four individual domains means to assume a non-cooperative opening/closing model for TRPV1 - I am not sure this has been thoroughly discussed in the literature yet, but with their system, the authors are in a unique position to address this point. Maybe this could be added in the discussion?

h) On p. 10, the authors compare their NMR-based secondary structure with available cryoEM data. May I suggest to add a word of caution here as the cryoEM structures often do not have sufficient resolution/lack representation of multiple conformers to unambiguously make statements about local structure. In contrast, I would encourage the authors to embrace the fact that NMR can yield propensities of secondary structure which may be much better suited to describe a molecule in solution than a symmetrized cryoEM/X-ray structure. Are the described differences in the TRPV1 cryoEM structures actual differences or just due to differences in (local) resolution or model building?

i) In Fig. 3a-d, the authors present a very thorough assessment of the protein at high temperature using state of the art methods such as advanced RDC analysis for mapping helix locations. Here the question arises how well the measured RDCs globally fit to structural models of the S1/S4 domain derived from the available TRPV1 structures and maybe even related TRPV channels? This is necessary to demonstrate that the overall structure and the relative orientation of helices with respect to each in the S1-S4 construct under NMR conditions is similar to that in the context of the full-length channel. Secondly, this could be used to identify structural differences between the NMR structure at high temperature and “ground state” cryoEM structures and allow conclusions about how similar the heat-activated structure is to e.g. the capsaicin or RTX bound structures.

j) Fig. 3 should be split in two parts – one that deals with the NMR-structure at high temperature and the comparsion with the cryo-EM structures and another one that aims at the NMR-characterization of temperature dependent conformational changes. Fig. 3b and c describe the S1/S4 secondary structure at high temperature and should be used for one figure. Figure 3a should be more clearly explained as highlighting the differences between the solution structure of the isolated S1-S4 at high temperature and the cryo-EM structure at low temperature and is a result of the data in panels b and c and should therefore follow them.
The other panels contain temperature-dependent data and describe the conformational changes seen by NMR when changing the temperature and should therefore presented in a separate figure.

k) Whether the temperature factors presented in panel 3c are well suited to describe a system that undergoes a temperature-dependent conformational change (including putative changes in hydrogen bonding) is not clear. Such a conformational change would imply that the temperature factors at low temperature for the same residue are different form the temperature factor at higher temperatures and thus the linear fitting of shift change vs. temperature used here to derive the temperature factor is no longer valid.

l) With regard to describing the temperature-dependent conformational changes by NMR, the authors miss a couple of opportunities here. First of all, even though it is not easily possible to derive backbone Ca, Cb or CO assignments at low temperature, the backbone amide assignments can be transferred to the low temperatures as already done by the authors. Then it is straightforward to compare the amide chemical shifts at low temperature with the respective random coil values and gather direct evidence for e.g. helix (un)raveling etc by comparing with the current panel 3b. To more sensitively describe changes in solvent exposure at the different temperatures, solvent PREs induced by gadolinium chelates should be measured (see e.g. work by Madl and coworkers). Can RDCs be measured at lower temperature? This additional experimental information would significantly strengthen this part of the paper and contribute to a more detailed understanding of conformational changes induced by temperature change.

m) Regarding the R557A mutant – the HSQCs of the Wt and the mutant S1/S4 should be directly compared to assess how many structural changes derive from the mutation within this domain. This is particularly relevant with regard to residue Y554, which should undergo shift changes when R557 is mutated. The authors are thus in the unique position to provide evidence in solution for an important coupling mechanism.

n) Similarly, the authors should provide the NMR titrations and chemical shift analyses (similar as in Fig. 1 for the wt) of the R557A mutant with capsaicin. The R557A mutant in electrophysiology shows no more response to capsaicin and the assumption is that coupling between S1/S4 to the pore domain is mediated via R557. But this suggests that capsaicin binding to S1/S4 R557A should be relatively unaffected? This should be shown.

**Author rebuttal, first version:**

**Point by point response to the Reviewers’ comments.**

We sincerely appreciate the careful, thoughtful, and constructive suggestions by both reviewers. On account, we have made many changes and enhancements to the manuscript. Below please find our responses to the suggestions and critiques. The original reviewers’ comments are in black text and our response in **blue**. In the revised manuscript and supplementary information, we have highlighted significant changes from the original documents in yellow.

Among the changes we have made include significantly strengthening the data supporting that R557 functions as a coupling mutation. The new data include the requested pH data, were we show that R557A is activated by protons with similar sensitivity as WT, and collecting an additional NMR titration of R557A that shows that the mutant does not alter capsaicin binding affinity. More specifically, additional data and figures included in the revised manuscript include:

Fig. 3c: H^N^ Δδ at 20 °C and corresponding secondary structure analysis.

Fig. 5f: Overlay of the WT and R557A HSQC spectra to support disruption of the cation-π

interaction.

Fig. S4: Capsaicin induced chemical shift perturbation mapped to the 5irz cryo-EM S1-S4

domain structure.

Fig. S6f: Analysis of all temperature-dependent peak height data.

Fig. S7: Simulations of the impact of temperature on relative peak height and related

parameters.

Fig. S9: Correlation of experimental RDC values with those predicted from 5IRZ.

Fig. S10: A plot of all amide proton temperature coefficient data and residual chemical shift

analysis.

Fig. S12e: Binding isotherms of WT and R557A which shows that the mutant binds capsaicin

with similar affinity.

Fig. 13: Electrophyiologcal characterization of R557A, WT, and empty vector, which show that

R557A is a functional channel activated by protons and control experiments which

indicate the channel trafficks.

Beyond the figures above, we include two new tables (Tables 3 & 4) and discussion of the new data and analysis. We also note that we identified a typographical error associated with the fluorescence ΔH measurements and corrected the erroneous value from ±9 to the correct value ±5 kcal/mol. In addition, we have made a number of grammatical and stylistic changes to streamline readability. Thank you again for your thoughtful analysis of our science.

**Reviewers' comments:**

*Reviewer #1 (Remarks to the Author):*

In this manuscript, Kim et al. use biophysical methods (solution NMR, Far-UV CD, and intrinsic fluorescence) to study temperature-dependent conformational changes in the S1-S4 domain of the heat-activated cation channel TRPV1. They find that this isolated region undergoes a temperature-dependent transition that is consistent with the temperature dependence of gating in the intact channel. Overall, this is an interesting study that provides molecular and biophysical details regarding the structural changes in a functionally important region of the channel. However, the conclusions that can be drawn from this study regarding the gating of the intact channel are limited, as direct evidence that the observed conformational change is actually coupled to opening of the channel is lacking. The functional patch-clamp data have their limitations, and do not allow strong conclusions.

Specific points

1) The authors use the TRPV1-R557A mutation, claiming that the mutated residue is essential for coupling the S1-S4 domain to the gate. Then, the authors show that this mutant cannot be activated by heat or capsaicin, and attribute this to the deficient coupling. However, there is no evidence that the mutated channel is active at all -

both tested stimuli are negative, and the currents shown in Figure 4a do not look at all like TRPV1-mediated currents. In the absence of any evidence that the mutant channel is correctly expressed in the membrane and can be activated, these results are meaningless.

We appreciate the frankness of the comment and subsequent suggestions below. We note that the original idea of R557 being important to coupling vanilloid binding (and heat activation) to gating is not a new idea, and, as far as we are aware at least in the context of TRPV1, originates from work by Vlachova and coworkers (JBC, 2010, v285, pp41455-41462), where they proposed both a cation-π interaction between Y554 and R557 and an interaction between R557 and E570. These studies were done prior to the seminal TRP channel structural work, which started to appear in 2013. In support of the Vlachova data, a second wave of truncated rTRPV1 structures by Julius, Cheng, and coworkers (Nature, 2016, v534, pp347-351), identified a structural rearrangement that includes R557 and E570 interacting upon vanilloid activation (c.f. Fig 5 in their paper). While not mentioned in their studies, analysis of their structures (PDB IDs: 5IRZ and 5IRX) show that the predicted cation-π interaction is likely in the resting/apo structure while absent in the activated structure, as we show in our Figure 6. Also as we pointed out in our manuscript, results from cryo-EM structural studies of mTRPV3 support the role of this coupling/switch mechanism. Sobolevsky and coworkers (NSMB, 2019, v26, pp994-998) make a mutation at the equivalent position of Y554 in TRPV1 (TRPV3 Y564A), which results in a mutant channel that they describe as “highly sensitized” (i.e., significantly increased basal open probability) but turns out to be generally temperature-insensitive, they report a Q10 of ~1.2. Our original manuscript depended primarily on the previously published functional data, the lack of responses to heat and capsaicin by R557A we presented in our manuscript, and the NMR-thermodynamic data of the isolated R557A hV1-S1S4 domain, which show that the mutant has similar thermosensitivity to the WT channel (except for a few kcal/mol which we attribute to the loss of the cation-π interaction between Y554 and R557).

With this in mind, both reviewers wanted this point strengthened and we now do so in the revised manuscript. As described below, we now include both electrophysiology data that indicates the R557A trafficks to the membrane, and is a pH-sensitive functional channel with proton sensitivity that reflects the WT pH sensitivity. Similary, as requested by reviewer 2, we also now include NMR binding data for R557A, to show that the mutant binds capsaicin. These outcomes are more fully described below.

The reviewer notes that the currents in Fig 4a (now Fig. 5a in the revised manuscript) do not resemble the WT TRPV1 channel. We note that mutations often impact fundamental channel properties (such as activation, inactivation, etc.) which can change the form of the current traces. Based on our proposed mechanism, the R557A mutant prevents interactions that stabilize the resting state via cation-π interactions with Y554, and interactions that stabilize the vanilloid and heat active states, via electrostatic interactions with E570. On account, we are not surprised to see changes in the current traces. As suggested and described below we now include pH studies which are thought to have a different molecular mechanism, these additional data help support our mechanistic hypothesis, see point #3.

2) The authors use a K-based pipette solution (I suppose that 315 mM K+-gluconate is a typo). As a result, a significant part of the outward currents in HEK cells will be carried by endogenous K-channels, which explains the characteristics of the outward currents in Figure 4a.

Yes, 315 mM potassium gluconate was a typographical error, we intended to write 135 mM. The manuscript is updated, thank you for pointing out the error.

With regards to the use of K^+^-based pipette solution, we chose the pipette solution because high intracellular potassium concentrations generally reflects the intracellular condition found in biology. We also note that TRPV1 is an outward rectifying channel, which may indicate functional roles for TRPV1 in potassium flux. Beyond these facts, we agree there is potential for endogenous

K+ channels to convolute small currents on account of our pipette solution. However, we note that the human cytomegalorvirus (CMV) promoter we use is a very strong promoter in HEK293 cells and that TRPV1 will also pass potassium ions. As such given the CMV promoter and channel rectification, the TRPV1 currents should dominate those from endogenous K^+^ channels. This idea is supported by a comparison of our temperature-dependent TRPV1 data with other published TRPV1 thermosensitivy measurements (see Table 1). This comparison suggests that these data are not meaningfully impacted by our pipette solution. Similarly, with the newly included pH studies (see below), we arrive at canonical values for both WT and R557A TRPV1 proton sensitivity, which suggests that the impacts from the pipette solution are negligible. We also note that in unpublished data we have compiled for a manuscript under preparation, the *EC*50 of capsaicin using the same conditions we arrive at canonical potency that is statistically indistinguishable from other currently unpublished data we have generated with high-throughput calcium ion fluorescence assays (Fig. 1 below). So in our hands, the three major modes of TRPV1 activation are indistinguishable from what is reported in the literature with this pipette solution. Beyond that the R557A proton sensitivity reflects the WT channel. As such our data indicate that the pipette solution we use does not significantly alter the TRPV1 fucntional properties.

**[REDACTED]**

3) To address points 1 and 2, I suggest the authors perform experiments with a Cs-or Na-based pipette solution, and evaluate whether the mutant channel can be activated by other stimuli such as low pH.

These are important and relevant points. We believe that because we arrive at canonical values for TRPV1-dependent heat, proton, and capsaicin sensitivity, there is no compelling reason to redo all of the electrophysiology experiments with a different pipette solution. To address the concerns about the R557A mutant we have now performed a number of experiments to ensure that the R557A TRPV1 channel is functional. Importantly, as suggested, we performed a series of proton activation studies and find that R557A, despite low conductance, is activated by pH (*EC*50 ≈ 5.7, Supporting Fig. 13). As a positive control, we made the same proton measurements with the WT channel, which gives a similar proton *EC*50. We also include two additional measurements that support R557A being a functional channel and pH activated. First is that current levels of R557A TRPV1 transfected cells are statistically increased relative to cells transfected with the empty pIRES2 vector (e.g. no R557A TRPV1); these data support the pH studies and indicate that R557A TRPV1 does indeed traffick to the membrane and retains some of the canonical TRPV1 functional attributes. Second, empty vector-transfected cells do not show meaningful pH sensitivity; whereas, WT and R557A TRPV1 channels do. We note that in addition to the control electrophysiology studies that indicate the channel trafficks, we intended to perform traditional

trafficking measurements, similar to what we have done previously in TRPM8 studies (J. Biol. Chem. 2018, 283, 9423-9434), to validate that the mutant channel makes it to the membrane. However, we were unable to obtain the required antibody prior to our lab being shuttered by COVID-19 (it remains shuttered at the time of resubmission). Notwithstanding the lack of traditional trafficking studies, given that we show pH activation in the presence of the R557A TRPV1 channel, but not in its absence, and that transfection of R557A TRPV1 increases basal current density, these results clearly indicate the channel is functional and directly addresses the original critique about channel trafficking and hope that the reviewer does as well. The outcomes of the pH-dependent electrophysiology and related experiments are shown in the new Supplementary Figure 13.

4) As indicated by the authors, also other parts of TRPV1 have been implicated in heat sensing in other studies (basically every region of the channel has been proposed). Therefore, one could conclude that the temperature dependent transitions are not localised to a specific region (e.g. S1-S4), but occur all over the channel. If the authors want to propose that S1-S4 is somehow of special importance, further evidence is needed to show that specific mutations in this region, affecting the described structural transitions, affect heat-induced channel gating in a consistent manner.

We agree with this assertion and given the many papers that seem to over interpret TRPV1 thermosensing results, we prefer to be cautious and let our data speak for itself. Future studies on the cooperative nature of heat activation, thermodynamic investigations of the isolated pore domain, and other structural and biophysical studies will be required to unambiguously

untangle the exact contributions to heat activation from regions that allosterically modulate the response. In light of this, the strength of our current work is that we show through biophysical approaches that the isolated S1-S4 domain is folded and capsaicin binding competent and undergoes a significant temperature-dependent conformational change. We contextualize the magnitude of the conformational change and propose a coupling-based mechanism. Our study is distinct because it provides a testable hypothesis regarding both conformational changes and the coupling mechanism from the biophysical data and evidence that supports this mechanistic hypothesis, which in turn validates the biophysical data. Additionally, our temperature-dependent NMR data provides “leads” that can be tracked down and evaluated in future studies.

We also note that our manuscript importantly informs the TRP thermosensing field. For example, Swartz and co-workers have very nice and compelling data that suggest the pore domain is involved in thermosensing (see PNAS, 2018, 115, E317-E324). Our R557A electrophysiology data directly enrich their studies as this mutation is outside of the TRPV1 region they regard as heat-sensitive in the TRPV1/Shaker channel chimeras. Similarly, a recent cryo-EM TRPV3 structural study on temperature sensing (NSMB, 2019, 26, 994-998), suggests that one can access the TRPV3 temperature-activated state with the Y564A mutation. However, this is equivalent to Y554 in hTRPV1, and our studies suggest that this forms part of the cation-π switch with R557 and E570 (Fig. 6). Taken together, we prefer to be cautious and not over-interpret out data; notwithstanding, our study provides new pieces to the thermosensing puzzle, including an experimentally testable hypothesis.

*Reviewer #2 (Remarks to the Author):*

Kim and Sisco et al investigated the isolated TRPV1 ion channel S1-S4 transmembrane bundle and its response to temperature and capsaicin. This is a highly relevant question as TRPV1 is one of the best studied protein thermosensors, with an activation temperature of ~42C, yet the molecular details of thermosensing remain unclear. Previous work has suggested that the residues and/or region(s) required for thermosensing are localized in the transmembrane region.

The authors took advantage of the fact that the isolated TRPV1 S1-S4 transmembrane domain shows clear evolutionary relationships with the sensing domains of other ion channels. They purified

the isolated human TRPV1 S1/S4 domain and, using a combination of solution NMR, CD and fluorescence spectroscopy, investigate its interaction with capsaicin and its conformational response to temperature. Notably, the authors present NMR spectra of this transmembrane domain in LPPG micelles which are of exceptional quality, thus allowing them to confidently assign the majority of the backbone resonances and to derive a number of other informative NMR parameters.

They present the first convincing data that the S1-S4 bundle undergoes well-defined local structural changes in solution in response to temperature that suggest a putative molecular mechanism for temperature sensing in this class of ion channels. These are highly interesting results and of broad relevance to a large number of researchers and therefore this manuscript is in general well suited for publication in Nature Communications if the authors address a few technical points:

a) Fig. 1b: Can the color code be changed, the apo state spectra (black) are almost impossible to see.

The superimposed HSQC of hV1-S1S4 capsaicin titration was revised as requested. Please see Fig. 1 for changes.

b) Fig. 1c: Can the authors please clarify their color code (light/dark grey) in the figure legend for better readability?

Thank you for pointing out our oversight, this has been addressed in the Fig. 1 legend.

c) Capsaicin interaction: The authors observe that the residues previously identified to be relevant for capsaicin binding also interact with capsaicin in the context of the isolated S1/S4 bundle, but from the HSQC in Fig. 1d its apparent that a large number of peaks are affected by capsaicin, thsu suggesting that in addition to the residues named by the authors, also neighboring amino acids or even allosteric sites are affected. NMR is a particularly valuable technique to identify such effects. I therefore strongly recommend to plot the chemical shift pertubation on a structural model of the S1/S4 bundle. This will help the reader and may teach us something about the long(er) range effects of capsaicin binding.

As suggested we include the chemical shift perturbations (CSPs) mapped on the S1-S4 structure. This is now Fig. S4. Given that this is beyond the focus of our current manuscript, which focuses on temperature-dependent measurements, and that we have a manuscript in preparation, investigating and quantifying TRPV1 ligand binding we simply note, as the reviewer astutely points out, that capsaicin induces widespread CSP.

d) The temperature sensing experiments are very exciting. van Horn and colleagues present an elegant combination of electrophysiology with CD, Trp fluorescence and NMR spectroscopy to measure the temperature dependent transition between two states. Could the authors please also specifically mention the inflection point temperatures for ALL the different experimental approaches (also for CD and fluorescence) in the figure legend so that these differences can be discussed? It would help readability if these values were all presented in one place.

The mid-point values of activation from each method, which we call T50, for electrophysiology, CD, intrinsic fluorescence, and NMR, have been included in the main manuscript, and specifically aggregated in the legend for Fig. 2 for readability as suggested. While we note that they are generally close numerically, we hesitate to say too much about the meaning of these values because the inflection point informs something about the change in entropy convoluted with the change in enthalpy and so the meaning of this value is more complex. Moreover, we are not certain if it is anything more than coincidence that the T50 values from electrophysiology studies of the full-length channel and biophysical studies of the isolated hV1-S1S4 are numerically close, future experiments will be needed to validate the meaningfulness of these results.

e) In Fig. 2d, the conformational changes related to temperature sensing are demonstrated to manifest themselves in the sigmoidal character of the peak intensity vs temperature curve. This is

only shown for a single residue in Fig 2d and another one in the supplements. It would be nice to see more examples included in the supplementary materials.

Are there residues far away from the residues affected by heat-induced conformational changes that do not display a sigmoidal curve? These should be shown for comparison.

To address this comment, we extracted resonance intensities (peak height) of all 71 assigned resonances across the temperature range, normalized these values between 0 and 1, and plotted normalized resonance intensities against temperature. This is now included in Supplementary Figure 6 as panel (**f**) with G548 from Fig. 2d highlighted. As shown in the figure, the majority of resonances demonstrate two-state behavior. We attempted to identify local changes by mapping the apparent ΔH from these measurements on a structural model. However, we did not identify any meaningful trends so we did not include nor discuss this in the revised manuscript.

f) Fig 2d: It is unclear what signal intensity refers to in this graph – is this signal height or the integral intensity? This needs to be clarified. In case it is the signal height, the authors should show a simulated graph of peak heights vs temperature for a rigid protein of the same size and correlation time since the linewidth for such a system also changes as a function of temperature and correlation times.

We apologize for the confusion. We have clarified the text to indicate that “signal intensity” is used synonymously as “peak height”. We used this measure because of resonance overlap is a serious complication at lower temperatures for our NMR data, thereby complicating the use of peak width. To address this concern, in addition to clarifying our language, we include a new section in the Supplementary Information Methods titled “Simulation of the temperature dependence of NMR peak height”, which describes the Matlab simulations we carried out and parameters used (Table 4), with the outcomes that show the predicted temperature dependence of the viscosity of water, the correlation time, the transverse relaxation rate, the peak width, and ultimately the peak height. As requested, these simulated values are now shown in Supplementary Figure 7 and represent a rigid protein with the hydrodynamic size of the hV1-S1S4. We note that the outcomes of these simulations support the two-state transition we detect with NMR, CD, and fluorescence.

g) Dividing the DH values (in Fig 2f) by 4 to derive the contributions of the four individual domains means to assume a non-cooperative opening/closing model for TRPV1 - I am not sure this has been thoroughly discussed in the literature yet, but with their system, the authors are in a unique position to address this point. Maybe this could be added in the discussion?

As we indicated above and given the many contradictory studies in TRP channel thermosensing, we prefer to be cautious in our data interpretation. We have planned future experiments that should inform to what extent the S1-S4 domain motions are cooperative, but these are beyond the scope of the current study. In the discussion section (fourth and fifth paragraphs) of our initial draft we attempted to address this exact issue. A few excerpts are reproduced below with enhancements added in the revised manuscript high-lighted in yellow:

“*Putting hV1-S1S4 thermosensitivity value into the context of the full-length TRPV1 channel will require future studies.”... “One challenge with interpreting the thermosensitivity of the isolated hV1-S1S4 relative to the tetrameric TRPV1 in biological conditions is that the cooperativity between monomers during heat-sensing and activation in TRPV1 remains unclear. For hV1-S1S4, which represents ~18% of the channel by mass, could potentially contribute the majority of the thermodynamic driving force (>80%) should the ΔH values of the isolated hV1S1-S4 be additive (non-cooperative) in nature. On the other hand, if S1-S4 domains allosterically couple very efficiently (fully-cooperative) in tetrameric channel state, then this domain might contribute as little as ~20 kcal/mol, or ~20% of the thermosensing driving force, to TRPV1 thermosensitivity.”*

And later (bottom of the fifth Discussion paragraph:

*“Further studies are required to better contextualize our thermodynamics studies with regards to the full-length channel and to decipher and quantify the cooperativity between membrane domains. If rat TRPV1 vanilloid ligand activation serves as a guide, concatemer studies have shown that a single capsaicin binding event can fully open the channel (i.e., highly-cooperative); on the other hand, equivalent proton activation studies, show that full activation requires all four TRPV1 proton-sensors and is generally non-cooperative.”*

h) On p. 10, the authors compare their NMR-based secondary structure with available cryoEM data. May I suggest to add a word of caution here as the cryoEM structures often do not have sufficient resolution/lack representation of multiple conformers to unambiguously make statements about local structure. In contrast, I would encourage the authors to embrace the fact that NMR can yield propensities of secondary structure which may be much better suited to describe a molecule in solution than a symmetrized cryoEM/X-ray structure. Are the described differences in the TRPV1 cryoEM structures actual differences or just due to differences in (local) resolution or model building?

This is an excellent point. We are also happy to fully embrace the multifaceted utility of NMR, and in no means intend to diminish the capability of NMR to identify secondary structure. As the reviewer notes, interpretation of secondary structure by cryo-EM is complicated by a number of factors including resolution and the potential for ensembles of structural states. We still think there is value in the comparison and we report values from the RCSB as calculated by the DSSP algorithm, which we believe is the long-term gold-standard to identify secondary structure from structures. We intend that these comparative studies help to contextualize our NMR data. Additional analysis at 20 °C, as suggested by the reviewer (point L below), also helps clarify the apparent similarities and differences between our solution and temperature-dependent data and from what we can gather from the existing cryo-EM structures. We are willing to modify the manuscript as the reviewer might suggest, but opine that the comparisons have inherent value to inform potential differences that might be exploited with further experimental interrogation.

i) In Fig. 3a-d, the authors present a very thorough assessment of the protein at high temperature using state of the art methods such as advanced RDC analysis for mapping helix locations. Here the question arises how well the measured RDCs globally fit to structural models of the S1/S4 domain derived from the available TRPV1 structures and maybe even related TRPV channels? This is necessary to demonstrate that the overall structure and the relative orientation of helices with respect to each in the S1-S4 construct under NMR conditions is similar to that in the context of the full-length channel. Secondly, this could be used to identify structural differences between the NMR structure at high temperature and “ground state” cryoEM structures and allow conclusions about how similar the heat-activated structure is to e.g. the capsaicin or RTX bound structures.

As suggested we went through and compared our experimental ^1^H-^15^N RDC values with predicted values from five cryo-EM structures, three rat TRPV1 and two mouse TRPV3 structures using the software PALES. This new analysis has been included as Supplementary Figure 9 and Supplementary Table 3. We note that the outcomes of this analysis are mixed. First there is a clear correlation between measured and predicted RDCs, indicative that the overall folds (structures) are related between the isolated S1S4 domain and that in the full-length channel. However, the correlations are generally low as might be expected given the resolution of the cryo-EM structures (≥ 3 Å), the gap(s) in primary sequence between human TRPV1 and rat TRPV1/mouse TRPV3 (which we corrected for in our analysis), errors from the addition of protons to the cryo-EM structures, and a number of other factors. Thus, beyond showing that our isolated domain reflects that of the cryo-EM structures, we are unable to identify structural differences between cryo-EM and NMR conformations apart from the dipolar wave analysis initially reported. We have discussed these outcomes in the manuscript.

j) Fig. 3 should be split in two parts – one that deals with the NMR-structure at high temperature and the comparsion with the cryo-EM structures and another one that aims at the NMR-characterization of

temperature dependent conformational changes. Fig. 3b and c describe the S1/S4 secondary structure at high temperature and should be used for one figure. Figure 3a should be more clearly explained as highlighting the differences between the solution structure of the isolated S1-S4 at high temperature and the cryo-EM structure at low temperature and is a result of the data in panels b and c and should therefore follow them. The other panels contain temperature-dependent data and describe the conformational changes seen by NMR when changing the temperature and should therefore presented in a separate figure.

As requested, we have divided the data into two separate figures. Given the suggestion below (point L) to include data/analysis at lower temperature, the updated Figure 3 now includes the suggested division of data supplemented with a newly added secondary structure characterization at 20 °C. As requested Figure 4 has the temperature-dependent NOE and PRE data.

k) Whether the temperature factors presented in panel 3c are well suited to describe a system that undergoes a temperature-dependent conformational change (including putative changes in hydrogen bonding) is not clear. Such a conformational change would imply that the temperature factors at low temperature for the same residue are different form the temperature factor at higher temperatures and thus the linear fitting of shift change vs. temperature used here to derive the temperature factor is no longer valid.

This is an interesting point and we are unfamiliar with a similar situation in the literature. Generally the major component of the slope of HN temperature factor has been attributed to thermal expansion reflected in hydrogen bonding. Others have leveraged the slight nonlinearity in some HN temperature factors to provide insight into excited conformational states by taking the residuals from the linear fit and fitting the residuals to a quadratic equation ( = ^2^ + + ) to provide a framework whereby the sign (and to some extent, the magnitude) of the quadratic coefficient, “*a*” gives rise to information about the energetic differences between states and if the excited state is more or less folded. Others have published both temperature coefficients (i.e. the slope of the HN temperature dependence) and the residual chemical shift analysis (fitting the residuals to a quadratic equation) even though there would be fluctuations between conformational states (and presumably corresponding changes in hydrogen bonding). We feel this is acceptable because the dominant factor for the temperature dependence of HN chemical shift is thermal expansion which has been correlated with hydrogen bonds. This linearity is well reflected from the quality of the linear fits (~1% error in our case). On the other hand, the small deviations from linearity that we and other see in the residuals are on the order of ~3% of the magnitude of the temperature coefficient which suggests that the temperature coefficients in our case reflect something about the general hydrogen-bonded state of the protein.

As a consequence of this comment/suggestion, we carefully analyzed the residual chemical shift analysis. As the reviewer suggests, our situation is unique in that instead of potentially accessing excited conformational states from variable temperature, we are effecting a temperature-dependent conformational change (as seen in the NMR, CD, and fluorescence data). The residual analysis identifies a new phenomenon, that to our knowledge has not been reported in the literature previously. To date, published residual chemical shift analysis has always been either random or quadratic in nature. The hV1-S1S4 residual analysis has a cubic form. As we now discuss in the manuscript, we conclude that the cubic polynomial shape supports that there is a temperature-dependent conformational change. This is extensively discussed in the revised manuscript (see pgs 29-30 for the most thorough with corresponding references to support our assertions), and we have included a new Supplementary Figure 10, which shows all the primary temperature coefficient data and the resulting linear fit, in panel (**a**), and plots of the corresponding residual chemical shift data and cubic fits in panel (**b**). We note that we intend to follow up on the novel residual chemical shift outcomes in future efforts.

If the reviewer strongly disagrees with us on this point, we are willing to remove the temperature coefficient data from the manuscript ,as it is not a central point, but provides redundancy for other data and analyses.

l) With regard to describing the temperature-dependent conformational changes by NMR, the authors miss a couple of opportunities here. First of all, even though it is not easily possible to derive backbone Ca, Cb or CO assignments at low temperature, the backbone amide assignments can be transferred to the low temperatures as already done by the authors. Then it is straightforward to compare the amide chemical shifts at low temperature with the respective random coil values and gather direct evidence for e.g. helix (un)raveling etc by comparing with the current panel 3b. To more sensitively describe changes in solvent exposure at the different temperatures, solvent PREs induced by gadolinium chelates should be measured (see e.g. work by Madl and coworkers). Can RDCs be measured at lower temperature? This additional experimental information would significantly strengthen this part of the paper and contribute to a more detailed understanding of conformational changes induced by temperature change.

As suggested we have now analyzed our amide proton chemical shifts at 20 °C and include that data and analysis in Figure 3C. The data show a handful of important changes in secondary structure that we now discuss in the manuscript. We had previously tried to obtain RDC data at 20 °C but the S/N and resolution were limiting. We had hoped to attempt the suggested Gd-DTPA paramagnetic relaxation wave experiments; however, we prioritized the suggested NMR binding measurements (point N below) and our lab (and the NMR facility) was shutdown do to COVID and currently remains closed. As such we have not been able for the past months to attempt these experiments. Because the analysis of the amide proton Δδ analysis at 20 °C gives redundant information with the suggested Gd-DTPA experiments we hope that this satisfies the reviewer’s suggestion.

h) Regarding the R557A mutant – the HSQCs of the Wt and the mutant S1/S4 should be directly compared to assess how many structural changes derive from the mutation within this domain. This is particularly relevant with regard to residue Y554, which should undergo shift changes when R557 is mutated. The authors are thus in the unique position to provide evidence in solution for an important coupling mechanism.

As suggested, we now include an overlay of the R557A and WT NMR spectra as shown in Fig. 5f. We note that differences surrounding Y554 are detected which we interpret as supporting the cation-π dependent coupling mechanism.

i) Similarly, the authors should provide the NMR titrations and chemical shift analyses (similar as in Fig. 1 for the wt) of the R557A mutant with capsaicin. The R557A mutant in electrophysiology shows no more response to capsaicin and the assumption is that coupling between S1/S4 to the pore domain is mediated via R557. But this suggests that capsaicin binding to S1/S4 R557A should be relatively unaffected? This should be shown.

As requested, we performed an NMR titration of capsaicin binding to the R557A hV1-S1S4. As expected, the outcomes indicate that R557A does not impact binding when the NMR data is analyzed either globally with principal component analysis (i.e., TRENDnmr, data shown in Fig. 2 which we intend to publish separately as part of a thorough TRPV1 binding study) nor when looking at specific key capsaicin resonances. We now include a comparison between R557A and WT of capsaicin binding isotherms for T550, a key capsaicin interacting residue, in Supplementary Fig. 12e and note that they are not meaningfully different. In support of this general idea and as suggested by reviewer 1, we also include new electrophysiology data which indicate that the R557A hTRPV1 is a functional proton sensing channel also supporting the mechanistic hypothesis we propose.

**[REDACTED]**

Again, thank you to both reviewers for their suggestions. Their recommendations have enhanced our manuscript.

**Reviewer comments, second version:**

Reviewer #1 (Remarks to the Author):

The authors have adequately addressed my concerns. The new data with pH activation of the R577A mutant convincingly show that this is still a functional channel, which was my main concern with the initial submission.


Reviewer #2 (Remarks to the Author):

The authors adressed all my concerns very well and I fully support publication of this nice paper. It is obvious that the authors underwent a tremendous effort for these revisions in particular during these difficult times. I think this was a nice example of authors and reviewers working together towards a common goal and I am looking forward to reading about the unexpected behavior of residual chemical shifts in a temperature-sensing protein in the near future.

The only small thing I would request before publication is to expand the legend in Figs. 3a-c for for the uninititated reader to state what the black spheres and red/blue lines represent.
P.18, bottom "has a consensus value is in the realm" - grammar?

**Author rebuttal, second version:**

**Reviewer #1 (Remarks to the Author):**

**The authors have adequately addressed my concerns. The new data with pH activation of the R577A mutant convincingly show that this is still a functional channel, which was my main concern with the initial submission.**

**Reviewer #2 (Remarks to the Author):**

**The authors adressed all my concerns very well and I fully support publication of this nice paper. It is obvious that the authors underwent a tremendous effort for these revisions in particular during these difficult times. I think this was a nice example of authors and reviewers working together towards a common goal and I am looking forward to reading about the unexpected behavior of residual chemical shifts in a temperature-sensing protein in the near future.**

**The only small thing I would request before publication is to expand the legend in Figs. 3a-c for for the uninititated reader to state what the black spheres and red/blue lines represent.**

**The following is now included in the Fig 3 legend:**

***“****In panels a-c, black circles are individual data points and are superimposed with fits of the* ***data to an α****-helical periodicity (red or blue lines respectively).****”***

**P.18, bottom "has a consensus value is in the realm" - grammar?**

We changed the mentioned text to the following:

**“...TRPV1 has a consensus value** is in the realm of ~100 kcal mol-1

**Reviewer comments, third version:**

**Author rebuttal, third version:**
